# Modeling immunotherapies in live 3D human cancer tissue bioreactors

**DOI:** 10.7150/thno.118298

**Published:** 2026-01-14

**Authors:** Yizheng Zhang, Ivan Foth, Ahmad Makky, Philip Bucher, Melanie Grimm, Peter-Martin Bruch, Ilona Hagelstein, Sascha Dietrich, Josef Leibold, Lukas Flatz, Judith Feucht, Sven Becker, Christian M. Schürch

**Affiliations:** 1 Department of Pathology and Neuropathology, University Hospital and Comprehensive Cancer Center Tübingen, Germany.; 2 Cluster of Excellence iFIT (EXC 2180) "Image-guided and Functionally Instructed Tumor Therapies", University of Tübingen, Germany.; 3 Department of Pediatric Hematology and Oncology, University Children's Hospital Tübingen, Germany.; 4 Department of Hematology, Oncology and Clinical Immunology, University Hospital Düsseldorf, Germany.; 5 Department of Medicine V, Hematology, Oncology and Rheumatology, University of Heidelberg, Germany.; 6 Spatial & Functional Screening Core, Center for Integrated Oncology Aachen-Bonn-Cologne-Düsseldorf, Düsseldorf, Germany.; 7 Department of Hematology and Oncology, University Hospital Düsseldorf, Düsseldorf, Germany.; 8 German Cancer Consortium (DKTK), partner site Tübingen, a partnership between DKFZ and University Hospital Tübingen, Germany.; 9 Clinical Collaboration Unit Translational Immunology, Department of Internal Medicine, University Hospital Tübingen, Germany.; 10 Department of Medical Oncology and Pneumology, University Hospital and Comprehensive Cancer Center Tübingen, Germany.; 11 Department of Dermatology, University Hospital and Comprehensive Cancer Center Tübingen, Germany.; 12 Department of Otorhinolaryngology, Head & Neck Surgery, University Hospital and Comprehensive Cancer Center Tübingen, Germany.

**Keywords:** perfusion bioreactor, 3D tissue culture, immune checkpoint inhibitor, CAR T cells, CODEX multiplexed fluorescence microscopy.

## Abstract

**Background:** Cancer immunotherapies have shown remarkable efficacy in advanced malignancies, yet many patients remain unresponsive. This variability, along with concerns about adverse effects and healthcare costs, highlights the need for predictive biomarkers and physiologically relevant cancer models to forecast individual treatment responses. Existing systems inadequately recapitulate the human tumor microenvironment (TME), which is essential for understanding immune-tumor interactions and treatment efficacy. Here, we developed an *ex vivo* 3D human tissue culture model that preserves the native TME for functional immunotherapy testing. Such a short-term culture platform also supports functional precision medicine by enabling rapid *ex vivo* assessment of therapeutic responses to guide clinical decisions.

**Methods:** Fresh, intact human lymph node (LN) tissue pieces were cultured in optimized perfusion bioreactors for three days, during which CAR T cell therapies and antibody-based treatments were administered. Post-culture analyses were performed using flow cytometry, histology, and multiplexed fluorescence microscopy.

**Results:** The bioreactor system significantly improved tissue viability compared to traditional plate cultures. Novel CAR T cells with enhanced PI3K signaling exhibited superior tissue infiltration but showed comparable cytotoxicity to conventional CAR T cells. Pembrolizumab, a PD-1 inhibitor, significantly reduced lymphoma and melanoma cell viability without affecting benign LN tissues.

**Conclusions:** This optimized bioreactor culture system provides a robust platform for evaluating immunotherapy efficacy within a physiologically relevant TME. It offers valuable potential for advancing personalized treatment strategies, accelerating the understanding of immunotherapy mechanisms, and improving clinical outcomes.

## Introduction

Advanced-stage cancer remains a leading cause of mortality globally 1. While early-stage solid tumors are often amenable to surgical intervention, advanced-stage cancers and hematological malignancies generally require more comprehensive treatment approaches, such as chemotherapy, radiotherapy and other non-surgical modalities 2. However, many patients develop resistance to these treatments, or fail to respond from the outset 3. In such cases, immunotherapy has emerged as a promising alternative, offering hope for patients with refractory or resistant cancers 4.

The efficacy of immunotherapeutic approaches is fundamentally dependent on their ability to effectively trigger immune responses against malignant cells 5. Immunotherapies, such as immune checkpoint inhibitors (ICIs) and chimeric antigen receptor (CAR) T cells have shown remarkable success in the treatment of certain cancer types, leading to durable remissions and prolonged survival in responsive patients 6,7. However, like for many other treatment methods, a substantial proportion of patients either do not respond to these treatments or develop resistance over time 8,9. Furthermore, immunotherapies can cause severe adverse effects, including life-threatening autoimmune reactions 9,10. The high cost of these treatments poses an additional burden on both healthcare systems and patients 11.

Tumors consist not only of neoplastic cells but also a diverse array of immune cells, stromal cells, vasculature, protein factors, and extracellular matrix (ECM) components, collectively known as the tumor microenvironment (TME) 12. This complex milieu plays a central role in influencing cancer development and behavior. Immune cells, both innate and adaptive, infiltrate the TME and can exert cytotoxic or inhibitory effects on cancer cells 13. However, as cancer evolves, malignant cells develop mechanisms to evade immune surveillance, including the downregulation of tumor-associated antigen expression and recruitment of immunosuppressive cells, leading to impaired antitumor immune responses 14-17. Early studies have highlighted the prognostic value of immunophenotypic characteristics within the TME for cancer patients 12,18. These challenges underscore the necessity for predictive biomarkers and models to identify which patients are likely to benefit from immunotherapy, thereby minimizing unnecessary exposure to adverse effects and optimizing healthcare resources. Thus, understanding how immunotherapies impact the interactions between cancer cells and immune cells, as well as their ability to remodel the TME, is critical for evaluating therapeutic responses and developing new treatment strategies.

The development of more effective and more precise therapies, as well as improved predictive biomarkers of treatment response, is limited by the lack of culture systems adequately mimicking the architectural and cellular complexity of human cancer tissues. Over the past few decades, significant progress has been made in the development of *in vitro* cancer culture models aimed at capturing the intricate cellular and spatial heterogeneity exhibited by tumors. These models have transitioned from simple two-dimensional (2D) plate cultures to more sophisticated 3D systems. For example, spheroids, initially introduced in the 1970s, involve the aggregation of suspended cells to form anchorage-independent spheres that closely mimic the fundamental 3D structure of tumors 19. Spheroids have emerged as a straightforward and convenient 3D model widely utilized for *in vitro* cancer cell culture 19. Another promising 3D model is organoids, which entail embedding cells within a matrix enriched with ECM proteins and subjecting them to stimulation by a specific growth factor cocktail 20. This approach facilitates cell expansion and spontaneous formation of structures exhibiting characteristic features and resembling the original organ 21. However, these models possess inherent limitations that impede their ability to fully recapitulate therapy responses and capture the intricate interplay between tumor cells and the immune contexture. These limitations encompass the absence of physical perfusion, restricted heterogeneity, genetic stability issues, and the absence of crucial TME components including fibroblasts, immune cells, endothelial cells, and a functional vascular network 22,23. Therefore, there is a pressing need to explore further models that can provide a more comprehensive representation of tumor biology and enable more accurate investigation of therapeutic interactions.

Direct *in vitro* culture of patient-derived tissues preserves the cellular composition and spatial organization of the TME, facilitating individualized therapeutic assessments for immunotherapy 24,25. However, maintaining 3D tissue viability after excision is challenging, as conventional culture methods often struggle to provide sufficient nutrient and oxygen exchange, limiting the duration for experimental analyses. Some studies have attempted to mitigate tissue viability loss by using very small tissue fragments, but this approach may compromise their physiological relevance to the native tissue environment 24,25. Bioreactor systems, which offer enhanced circulation and nutrient delivery, have shown promise in addressing this issue. Various bioreactor types, including spinner flask, rotating wall vessel, microfluidic, and perfusion bioreactors, have been effectively applied in 3D tissue culture to support morphological and functional studies and improve therapeutic modeling 26-29.

In this study, we utilized 3D tissue perfusion bioreactors 29-31 to develop a lymphoid tissue immunotherapy model aimed at identifying responsive patients prior to treatment initiation. By recreating the lymphoid tissue microenvironment *in vitro*, this model enables the evaluation of lymphoid tissue responses to various immunotherapies and facilitates the identification of biomarkers to stratify patients based on treatment sensitivity. Our optimized bioreactor culture system supports the viability of human lymphoid tissues, allowing precise evaluation of immunotherapy efficacy. Using this platform, we demonstrated pembrolizumab's selective cytotoxicity against lymphoma and metastatic cancer cells in lymph nodes (LNs) while sparing benign tissues. Cytokine analysis revealed pembrolizumab-induced increases in T cell activation markers, including IFN-γ, granzyme B, and perforin, which correlated with enhanced CD8⁺ T cell infiltration. Furthermore, our culture model revealed that CAR T cells with enhanced PI3K signaling exhibit superior tissue penetration compared to conventional CAR T cells, supporting the potential of balanced PI3K signaling in CAR T cell therapy. This bioreactor culture system represents a valuable platform for developing and testing personalized immunotherapies. By enabling patient stratification, reducing side effects in non-responders, and lowering healthcare costs, this approach has the potential to significantly improve patient outcomes. Additionally, our findings contribute to a deeper understanding of the factors influencing immunotherapy responses in lymphatic malignancies, supporting the development of novel treatment strategies.

## Materials and Methods

### Antibodies, reagents, equipment, buffers and culture medium

The source and detailed information of antibodies, reagents and equipment are shown in the key resources table (**Table S1**). The information on composition of buffers, working solutions, and culture medium are shown in **Table S2**.

### Sample acquisition

Human lymphoid tissue samples were freshly obtained from the University Hospital Tübingen and immediately processed. All patients gave written informed consent, and the study was approved by the local ethics committee (022/2021BO2). Patient sample cases and their pathological diagnoses are detailed in **Table S3**. Some samples were utilized in multiple experiments.

### Sample preparation and tissue culturing

**Figure 1:**
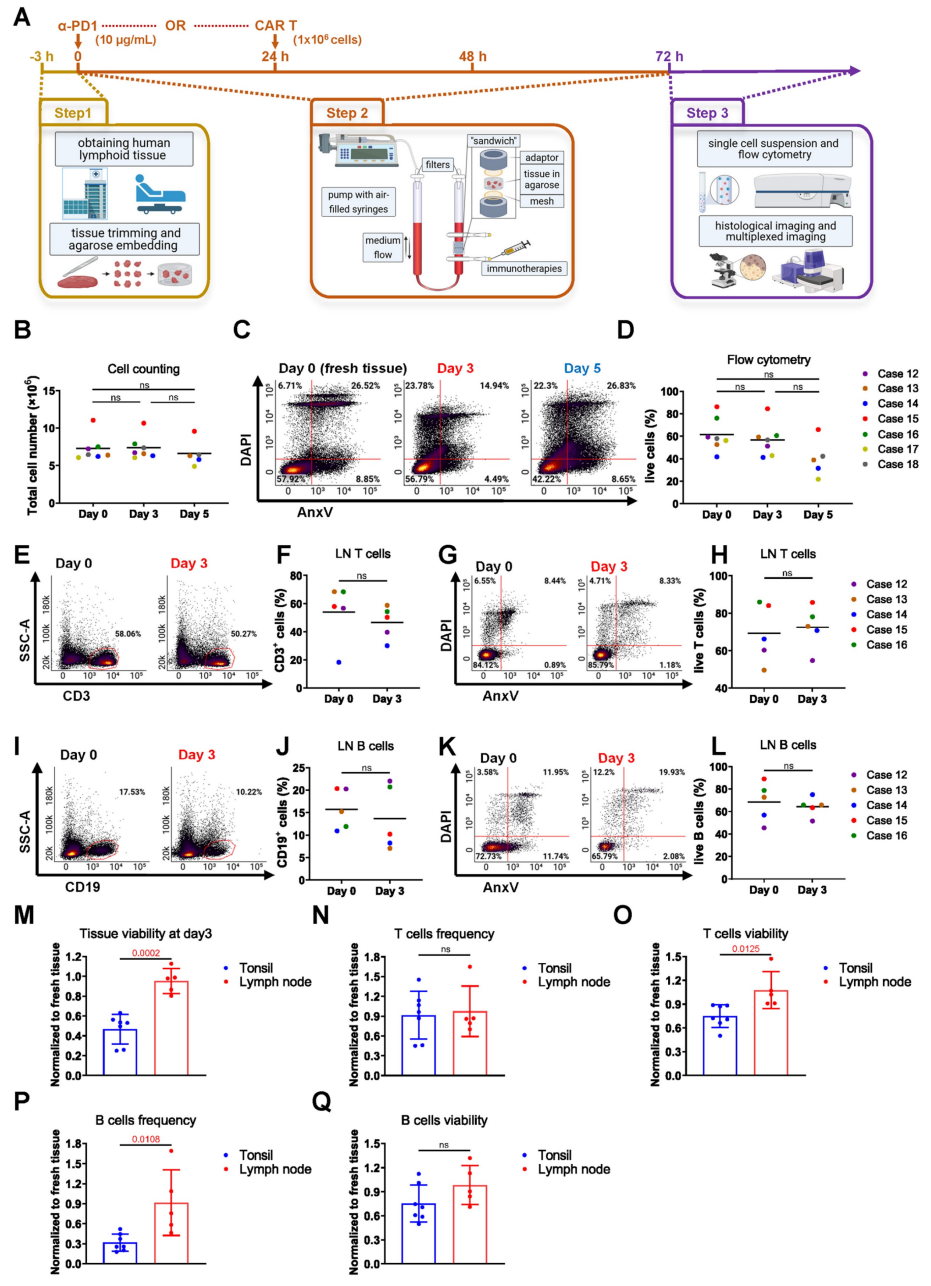
** Optimized bioreactor culture conditions on LN tissues. (A)** Schematic overview of human lymphoid tissue preparation, 3D perfusion bioreactor culture, immunotherapy dosing and application schedule, and subsequent tissue analysis. **(B)** Estimated total cell numbers in each bioreactor before and after culture based on cell counting (n = 5-7). **(C)** Representative flow cytometry plots and **(D)** viability analysis of LN tissues at day 0 and after 3-5 days of culture (n = 5-7). **(E-F)** T cell frequencies and **(G-H)** viabilities in LN cultures from day 0 to day 3 (n = 5).** (I-J)** B cell frequencies and (**K-L**) viabilities over the same culture period (n = 5). **(M)** Overall tissue viability, **(N)** T cell frequency, **(O)** T cell viability, **(P)** B cell frequency, and (**Q**) B cell viability were compared between LN and tonsil tissues, normalized to fresh tissue values (n = 5-7). Information on human sample cases is provided in **Table S3**. Statistics: **(B)** Kruskal-Wallis test with Dunn's multiple comparisons; **(D)** one-way ANOVA with Tukey's post hoc test; **(F)** Wilcoxon matched-pairs signed-rank test; other comparisons: unpaired or paired t-test as appropriate.

Fresh tissues were washed twice in EDTA-PBS buffer to eliminate any residual blood. Subsequently, a 3-4-millimeter-wide disc was carefully excised along the longest tissue axis, which was further subdivided into 3x3x3mm pieces. For each bioreactor, 4 tissue pieces were placed between 2 collagen sponges (Cellec Biotek) or embedded in 300 μL of 0.5% agarose solution (Biozym, 840004) at a temperature of 40 °C. The embedded samples were positioned between two adaptors within the chamber of the perfusion bioreactor (Cellec Biotek). Subsequently, a volume of 10 mL of the final culture medium was injected into the chamber through the valve. Following this, the bioreactors were connected to a syringe pump (Harvard Apparatus), and perfusion was performed in a cyclic manner using the following protocol: infusion of 3 mL at a constant rate of 1 mL/min, followed by a 5-second delay, withdrawal of 3 mL at 1 mL/min, another 5-second delay, and repetition of the cycle throughout the culture period. The bioreactor culture was maintained for a duration of 3 days at 37 °C and 5% of CO_2_ in an incubator. Conventional static plate culture was used as a comparison for tissue viability. In each experiment, tissues from the same patient were prepared as described earlier. Four tissue pieces were placed in 6-well tissue culture plates (Falcon, 3003055) with 3 mL of the same culture medium per well and cultured in the same incubator. Thereafter, the post-cultured samples were harvested. 2 pieces were embedded in paraffin and Tissue-Tek O.C.T. compound (Sakura, SA62550) for making paraffin and fresh-frozen tissue blocks and for histological stainings, respectively, and the other 2 pieces were used to make a single cell suspension for flow cytometry experiments (**Figure 1A and Figure S1A-D**).

### Single-cell suspension cell count and flow cytometry analysis

The harvested tissue pieces were further minced with a scalpel, grinded with a syringe plunger and filtered through 100 μm, 70 μm and 40 μm strainers (pluriSelect). 10 μL of each suspension was mixed with 10 μL trypan blue (Sigma-Aldrich, T8154) for counting the number of total cells, live cells and dead cells using a Neubauer chamber (Hecht Assistent). To estimate the total cell number per bioreactor, two out of four tissue pieces after culture were dissociated and counted individually. The total cell number was then extrapolated by multiplying the sum of this cell count by two, assuming similar cellularity across all tissue pieces. For cell counting of fresh cells before culture, two fresh tissue pieces were used instead. The rest of the sample was equally divided into polystyrene tubes (Falcon, 352058), and incubated with fluorophore-labeled primary antibodies and a DAPI/Annexin V cocktail (see **Table S1**) for 30 min at 4 °C. Annexin V binding buffer was used for washing and antibody dilution. Flow cytometry was conducted using a BD LSR II Flow Cytometer (BD Biosciences).

### Checkpoint inhibitor treatment

Lymphoid tissues were cultured in bioreactors as described above. On day 0 of culture, PD-1 inhibitor pembrolizumab (Selleckchem, A2005) was added to the culture medium at a final concentration of 10 µg/mL. Untreated tissues cultured under identical conditions served as controls. After 72 h, tissue samples were harvested for viability analysis and immune profiling. Flow cytometry was used to assess tissue viability markers (Annexin V, DAPI) and T cell markers. Fluorescence imaging was performed to evaluate tissue structure and pembrolizumab's effects on the TME (**Figure 1A**).

### Generation of CAR T Cells

CD19-CAR T cells were generated as previously described 32,33. Briefly, peripheral blood mononuclear cells (PBMCs) were collected via leukapheresis and activated with anti-CD3/CD28 antibodies (MACS GMP Pure, Miltenyi Biotec). Transduction was performed using the RV-SFG.CD19.CD28.4-1BBzeta retroviral vector (provided by Prof. Malcolm Brenner, Baylor College of Medicine, Houston, TX, USA), containing an anti-CD19 scFv (FMC63). Post-transduction, cells were cultured in medium supplemented with 10 ng/mL IL-7 and 5 ng/mL IL-15 (Miltenyi Biotec) for 13 days, with medium changes on days 7 and 10. Transduction efficiency was assessed by flow cytometry, and cells were cryopreserved after sterility confirmation.

### Generation of PI3K Enhanced CAR T Cells

PI3K enhanced CAR T cells were generated using base-editing methods (Bucher et al. 34). Briefly, the base editor plasmid pCMV-AncBE4max (Addgene #112095) was linearized with AgeI-HF (New England Biolabs, NEB, R3552) and transcribed *in vitro* using the T7 MEGAscript Kit (ThermoFisher, AMB13345). Transcripts were capped with CleanCap AG (Tebubio, N-7113), PolyA-tailed (ThermoFisher, AM1350), and purified via overnight LiCl precipitation (included in the T7 MEGAscript Kit). T cells were isolated, activated, and cultured as previously described 35. After 48 h, 3 million T cells were resuspended in 100 μL buffer T and electroporated with 10 µg of AncBE4max mRNA and 5 µg of each synthetic sgRNA (Integrated DNA Technologies, IDT) in IDTE pH 7.5 buffer (IDT, 11-05-01-05) using the Neon Transfection System (Thermo Fisher) with 1400 V, 3 pulses and 10 ms. Mock-edited T cells were electroporated with scrambled sgRNA sequence GCACTACCAGAGCTAACTCA. The E81K point mutation was induced with sgRNA sequence GCTCTTGCTGCTCCGCTGTC. Following electroporation, cells were rested in antibiotic-free X-Vivo15 medium (Lonza, BEBP02-061Q) supplemented with 5 ng/mL IL-7 and IL-15 for 24 h. Retroviral transduction of the RV-SFG-CD19-4-1BB-CD3ζ CAR construct was then performed as previously described 35-37,65. Transduced CAR T cells were rested for at least 48 h before use.

### CFSE labeling and CAR T treatment

The *CellTrace™* carboxyfluorescein succinimidyl ester (CFSE, ThermoFisher, C34554) dye was employed to enable the tracking of anti-CD19 CAR T cell localization. A total of 1 × 10⁶ T cells (non-transduced [NT] T cells, conventional CAR-T, or PI3K-CAR-T) were injected per bioreactor condition. While the exact number of cells in each lymph node explant varied, based on post-culture total cell counts (~7.5 × 10⁶ cells per bioreactor; see Figure 1B) and the average frequency of B cells (~15%) determined by flow cytometry, the administered CAR T cell dose corresponds to an estimated E:T ratio of approximately 1:1. Briefly, CAR T cell samples stored in liquid nitrogen were thawed in a 37 °C water bath, while freshly cultured CAR T cells were used directly. Following two washes with PBS, 1 μL of CFSE was added to each 1 mL of cell suspension, and the mixture was incubated at 37 °C for 10 min. The reaction was terminated by adding 100 μL of fetal calf serum, followed by a single PBS wash. Subsequently, the cells were resuspended in the final culture medium and introduced into the bioreactor system. The administration of CAR T cells took place 24 h after initiating the bioreactor culture and lasted for 48 h (**Figure 1A**).

### Agarose-coated plate co-culture assay

To evaluate the impact of agarose coating on CAR T cell viability and function, 24-well plates were either coated with 150 μL of 0.5% agarose (in PBS) or left uncoated. CAR T cells or NT T cells (4 × 10⁵ cells/well) were co-cultured with diffuse large B cell lymphoma cell lines SU-DHL-2 or TMD-8 as target cells (4 × 10⁵ cells/well) in bioreactor culture medium. Co-cultures were maintained for 48 h at 37 °C and 5% CO₂. After incubation, T cell viability and CAR T cell-mediated target cell killing were assessed by flow cytometry.

### Measurement of cytokines

The LEGENDplex™ Human CD8/NK Panel (13-plex, #741186) was used according to the manufacturer's protocol (BioLegend). Samples, standards, and assay buffer were added to a 96-well plate, followed by fluorescence-labeled beads specific to the target cytokines. The plate was incubated at RT for 2 h with gentle shaking to allow binding. After washing to remove unbound substances, detection antibodies and streptavidin-PE were added and incubated for 1 h at RT. The plate was washed again, and samples were resuspended in sheath fluid. Data were acquired using a FACS Canto II flow cytometer (BD Biosciences), and analyte concentrations were quantified using LEGENDplex™ data analysis software (BioLegend).

### Immunofluorescence (IF) staining and imaging

Tissue samples were embedded in Tissue-Tek O.C.T. compound, frozen, and sectioned at 5.0 µm using a cryostat (Leica, CM3050S) at -20 °C. Tissue sections were stored at -80 °C or used immediately for staining. Initially, sections were fixed in acetone (neoFroxx, LC-4916.2) for 10 min at RT. Subsequently, sections were fixed with 1.6% paraformaldehyde (PFA) in PBS for 10 min. Following fixation, sections were blocked with 5% bovine serum albumin (BioSell) in PBS at RT for 0.5 to 1 h to prevent non-specific binding. Subsequently, the primary antibodies were added to the slides and incubated for 1.5 h at RT. Fluorophore-labeled secondary antibodies were then applied to the slides and incubated in the dark for 1 h at RT. Following this, a 10 min staining with 0.5 µg/mL DAPI (BioLegend) diluted in PBS was performed to visualize the cell nuclei. Imaging was performed using an inverted fluorescence microscope (BZ-X810, Keyence) equipped with a 20x CFI Plan Apo λ objective (Nikon). DAPI, CFSE, PE, and APC were captured through the DAPI, GFP, TRITC, and Cy5 filters, respectively.

### Hematoxylin and eosin (H&E) staining

For each sample, one well-preserved 2.5 μm thick FFPE section was selected for staining. After baking in an oven at 70 °C for 1 h, slides were transferred to xylene for 3 × 10 min, followed by 2 × 3 min in 100% ethanol, 2 × 3 min in 95% ethanol, 3 min in 80% ethanol, 3 min in 70% ethanol, and finally 2 × 3 min in double-distilled water. Slides were then immersed in 100% isopropanol for 45 s, followed by hematoxylin (Sigma-Aldrich, 1.09253) staining for 45 s. After rinsing with warm water of 30°C for 45 s, eosin (Sigma-Aldrich, 318906) was applied for 30 s and rinsed with water for 15 s. Slides were re-immersed in isopropanol (SAV Liquid Production, 67-63-0) for 45 s, transferred to xylene for 45 s, and mounted with Cytoseal (Epredia, 8312-4) and coverslips (Marienfeld Superior, 0101222). To quantify the total tissue area and necrotic regions, we utilized QuPath (version 0.3.0) 38. The entire tissue area was initially identified using the 'Create Thresholder' command, while necrotic regions were manually delineated with the 'Brush' tool under the supervision of a board-certified pathologist (C.M.S).

### CODEX staining and imaging

CODEX staining and multicycle imaging was performed as described previously 39. For each sample, one well-preserved 5 μm thick section with intact and unfolded tissue was selected for further experiments. Fresh-frozen sections on 22 x 22 mm Poly-L-Lysine (Sigma-Aldrich) coated coverslips (Marienfeld Superior, 0101052) were dried at RT for 2 min, fixed in acetone for 10 min, and then dried again. A circle was drawn around each section using a UV-curable glue pen (Bondic VIKO UG). The sections were rinsed with S1 buffer for hydration and fixed with 1.6% PFA for 30 min. To reduce autofluorescence, sections were then incubated in bleaching solution (**Table S2**) twice between two LED Pads (Aibecy A5 Ultra Bright 25,000 Lux LED Pads) for a total of 90 min, with solution replacement after 45 min. After washing with S1 buffer for 2 x 2 min, the sections were blocked with CODEX blocking solution containing mouse and rat IgG (Biozol), salmon sperm DNA (ThermoFisher), and a mixture of non-fluorescent CODEX oligonucleotides (Biomers) for 1 h at RT. Primary antibody cocktails (**Table S1**) were added and incubated overnight at 4°C. Following 2 x 2 min washes in S2 buffer and fixation with S4 buffer containing 1.6% PFA for 10 min, the sections were subjected to ice cold methanol (Honeywell) for 5 min and fixed with freshly prepared 4 mg/mL bis(sulfosuccinimidyl)suberate (BS^3^, ThermoFisher) fixative in PBS for 20 min. After final PBS washes, sections were stored in buffer S4 until imaging. The detailed recipes for all the buffers mentioned above are provided in **Table S2**. CODEX multiplexed imaging was conducted using a Keyence BZ-X810 inverted fluorescence microscope equipped with a Nikon 20x CFI Plan Apo λ objective and a PhenoCycler system (Akoya Biosciences). The detailed imaging settings and data acquisition methods have been previously described 40,41. Briefly, the first and last imaging cycles were acquired without fluorescent oligonucleotides to capture background fluorescence, which was subsequently subtracted during image processing. Two markers per cycle were imaged using the TRITC and Cy5 channels across all fluorescence cycles, while Hoechst and CFSE were imaged with DAPI and GFP channels respectively, with 9 z-planes acquired per cycle at a step size of 1.5 µm. Images were acquired at 20× magnification with a 3 × 3 tile configuration to maximize tissue coverage. Exposure times were individually optimized for each marker. Raw data were processed using the CODEX Processor software (Akoya Biosciences), which performs background subtraction, generates high-resolution composite images, segments cells based on DAPI nuclear staining, and exports single-cell marker expression data as a CSV file. Overlay images were created using ImageJ (Fiji, version 1.53t). Cell annotation and single-cell x-y coordinate analysis were performed using QuPath (version 0.3.0). Immune cell annotation was performed using threshold-based detection (via the 'Classify' → 'Object Classification' → 'Create Single Measurement Classifier' commands in QuPath), with thresholds defined in consultation with the pathologist and standardized across all replicates within the same experimental group (see threshold values in figure legends). Cell-cell distance analysis was performed using a custom Python-based algorithm (**Supplementary Material 1**).

### Statistical analysis

Statistical analyses were conducted by using GraphPad Prism software 8.0.2 (GraphPad Software). T-tests, RM one-way ANOVA (with Tukey's post hoc test), and non-parametric tests (Wilcoxon signed-rank test, Friedman test or Kruskal-Wallis test with Dunn's post hoc test) were used to compare differences between samples, depending on data distribution and pairing. Normality was assessed prior to test selection. Effect sizes were reported using R squared, eta-squared (η²), Wilcoxon r, or Kendall's W, as appropriate. Details of all statistical tests, normality assessments, multiple comparison corrections, and effect size metrics are summarized in **Table S4 (Summary of Statistics)**. P < 0.05 was considered statistically significant.

## Results

### An optimized 3D perfusion bioreactor model to study human lymphoid tissues

3D perfusion bioreactors of tissue explants are important tools to mimic the TME 29-31,42. To study the lymphoma TME, we first optimized culture conditions using human lymphoid tissues from the Waldeyer's ring, including palatine tonsils (tonsils) and pharyngeal tonsils (adenoids), which are readily and abundantly available. In first experiments using the manufacturer's original protocol, in which tissues are cultured between two collagen sponges 43,44 (**see Methods and Figure S2A**), we observed poor viability and substantial efflux of immune cells from the tissue into the culture medium.

Agarose is a soft, thermosensitive, and biocompatible natural hydrogel that effectively mimics the cellular microenvironment 45. A gel consisting of 0.5% agarose has been widely used for the 3D culture of various tissue and cell types, including lymphoid tissue, providing an ideal supportive matrix 46-48. In our approach, we substituted the collagen sponges by embedding the tissue pieces directly into 0.5% agarose to improve tissue culture outcomes (**Figure 1A** and **Figure S2A**). For reference, we first quantified the estimated total number of cells in each bioreactor before and after culture (**Figure S2B**). Agarose optimization resulted in reduced cellular efflux into the medium, with a higher proportion of cells retained within the tissue (**Figure S2C**) and a significantly increased cell viability after 3 days of culture, as analyzed by trypan blue staining and flow cytometry (**Figure S2D-F**). H&E stainings of tissues post-culture further showed a trend towards reduced necrotic tissue areas in agarose-embedded vs. collagen sponge-embedded tonsil pieces (**Figure S2G-H**). As part of the optimization process, we also tested an alternative ECM matrix, Basement Membrane Extract (BME), for tissue embedding. However, BME did not improve post-culture tissue viability compared to the original setup (**Figure S2I**). Moreover, analysis of two major immune cell types, T cells and B cells, revealed significantly improved frequencies and viabilities with agarose embedding, also in comparison to fresh tissue directly after surgery (**Figure S3A-H**).

When trying to optimize immune cell viability in bioreactor cultures, we tested various cytokine combinations and observed no significant changes in T or B cell survival with interleukin (IL)-2, IL-7, IL-4, or IL-6 supplementation. However, a slight trend toward improved T cell viability with IL-2 and IL-7, along with their relevance for CAR T cell function, led us to include IL-2 and IL-7 in subsequent experiments (**Figure S3I-L**).

Additionally, we compared the 3D perfusion bioreactor culture setups with conventional, static culture of tonsil pieces in tissue culture plates, both with and without agarose embedding. Cell viability was significantly higher in the perfused, agarose-embedded bioreactors compared to sponge-embedded bioreactors or plate culture (**Figure S4A-C**), indicating that agarose embedding in conjunction with tissue perfusion is the optimal strategy for the 3D culture of intact, live lymphoid tissues. Although no visible signs of nutrient depletion were observed, we cannot exclude the possibility that the lower medium volume (3 mL in static culture vs. 10 mL in the bioreactor) may have contributed to reduced tissue viability.

Following the determination of the optimal configuration for lymphoid tissue culture, our bioreactor system was expanded from tonsil tissue to LNs. The estimated total number of cells in each bioreactor was first quantified before and after culture (**Figure 1B**). Flow cytometry analysis revealed sustained cell viability in LN tissues over a 3- to 5-day culture period, with noteworthy preservation of tissue viability on day 3, nearly matching that of freshly harvested tissue on day 0 (**Figure 1C-D**). Additionally, the frequencies and viabilities of CD3⁺ T cells **(Figure 1E-H)** and CD19⁺ B cells **(Figure 1I-L)** remained stable after 3 days of culture compared to fresh LNs.

We compared the viability retention among different lymphoid tissues in a bioreactor. To account for variability in baseline viability between samples, each cell frequency and viability measurement after culture was normalized to its corresponding fresh tissue from the same donor. LN tissues exhibited higher overall viability compared to tonsil tissues **(Figure 1M)**. Both tissue types maintained similar T cell frequencies **(Figure 1N)**; however, LN tissues demonstrated significantly greater T cell viability **(Figure 1O)**. Additionally, LN tissues preserved B cells more effectively, as indicated by higher B cell frequencies **(Figure 1P)** and a trend toward increased B cell viability **(Figure 1Q)**.

In summary, these data demonstrate that replacing the original collagen sponge with 0.5% agarose in a 3D perfusion bioreactor system significantly enhances lymphoid tissue culture. This modification reduces cellular efflux, increases cell viability, and surpasses the performance of conventional plate culture methods, particularly in the culture of LN tissue.

### Maintenance of diverse cell subtypes and tissue architecture in 3D perfusion bioreactors

**Figure 2:**
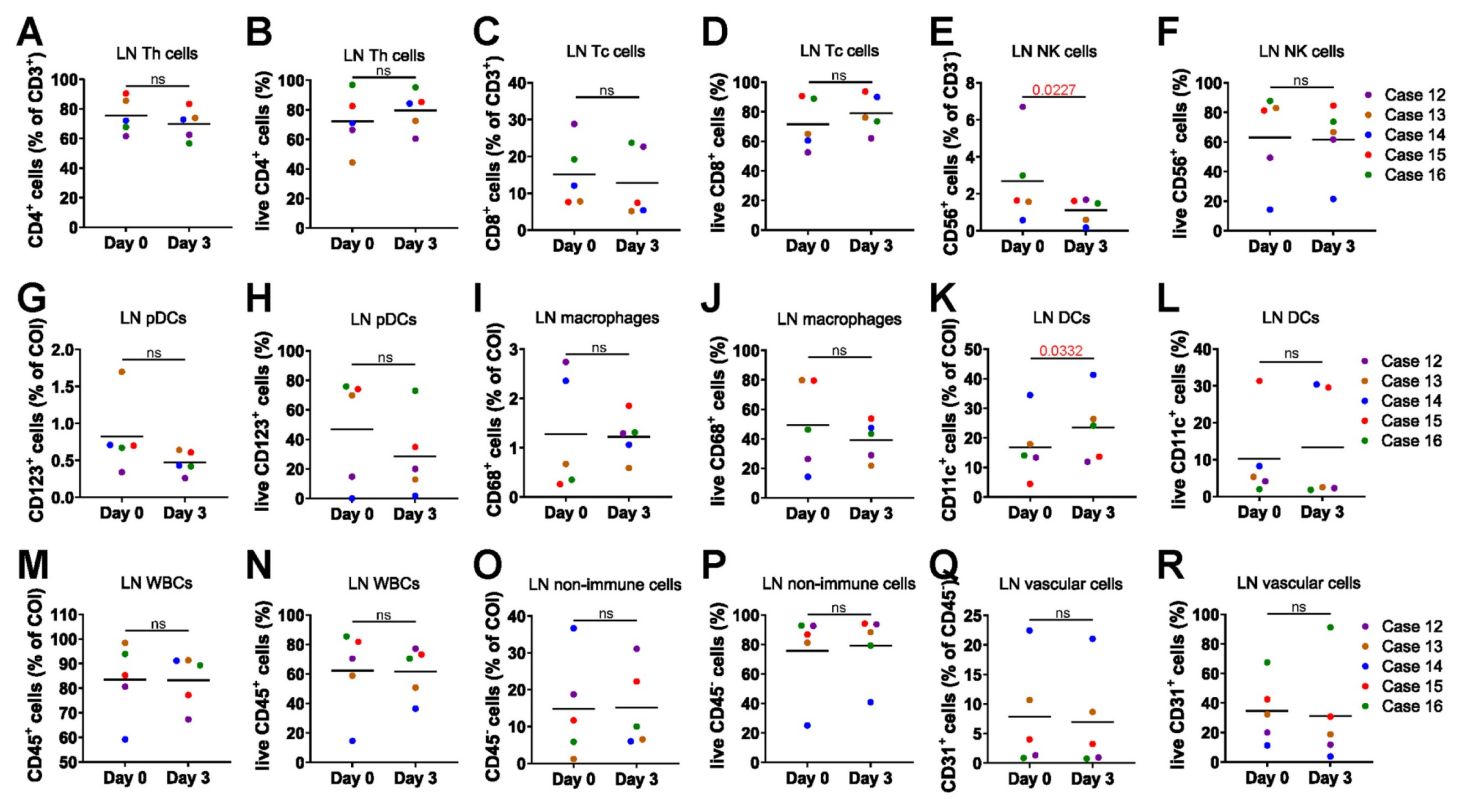
** 3D perfusion bioreactors sustain diverse lymphoid cell subsets.** Comparison of cell frequency and viability between day 0 fresh tissue and day 3 post-culture LN tissue (n = 5). **(A-B)** Frequency and viability of T helper cells. **(C-D)** Frequency and viability of cytotoxic T cells. **(E-F)** Frequency and viability of NK cells. **(G-H)** Frequency and viability of pDCs. **(I-J)** Frequency and viability of macrophages. **(K-L)** Frequency and viability of conventional DCs. **(M-N)** Frequency and viability of WBCs. **(O-P)** Frequency and viability of non-immune cells. **(Q-R)** Frequency and viability of vascular cells. Statistics: (L) Wilcoxon matched-pairs signed-rank test; other comparisons: paired t-test or ratio paired t-test as appropriate.

We performed a detailed analysis of the cell composition and viability of the cultured LNs using flow cytometry. Single-cell suspensions were stained with 3 distinct antibody panels. The gating strategy removed doublets and debris, isolating cells of interest (COI) and identifying populations by surface markers (**Figure S5A-C**). CD4⁺ T helper cells maintained consistent cell frequency and viability between day 0 fresh tissue and day 3 cultured tissue (**Figure 2A-B**). Similarly, CD8⁺ cytotoxic T cells showed no significant differences in cell frequency and viability between the two time points (**Figure 2C-D**). CD56⁺ natural killer (NK) cells showed a decrease in frequency but maintained stable viability (**Figure 2E-F**). CD123⁺ plasmacytoid dendritic cells (pDCs) (**Figure 2G-H**) and CD68⁺ macrophages (**Figure 2I-J**) also exhibited stable cell frequency and viability. A slight increase in the frequency of CD11c⁺ dendritic cells was observed; however, their cell viability remained unchanged (**Figure 2K-L**). CD45⁺ white blood cells (WBCs) (**Figure 2M-N**), CD45- non-immune cells (**Figure 2O-P**) and CD31⁺ vascular cells (**Figure 2Q-R**) maintained their frequency and viability, showing no significant differences from pre-culture fresh tissue.

**Figure 3:**
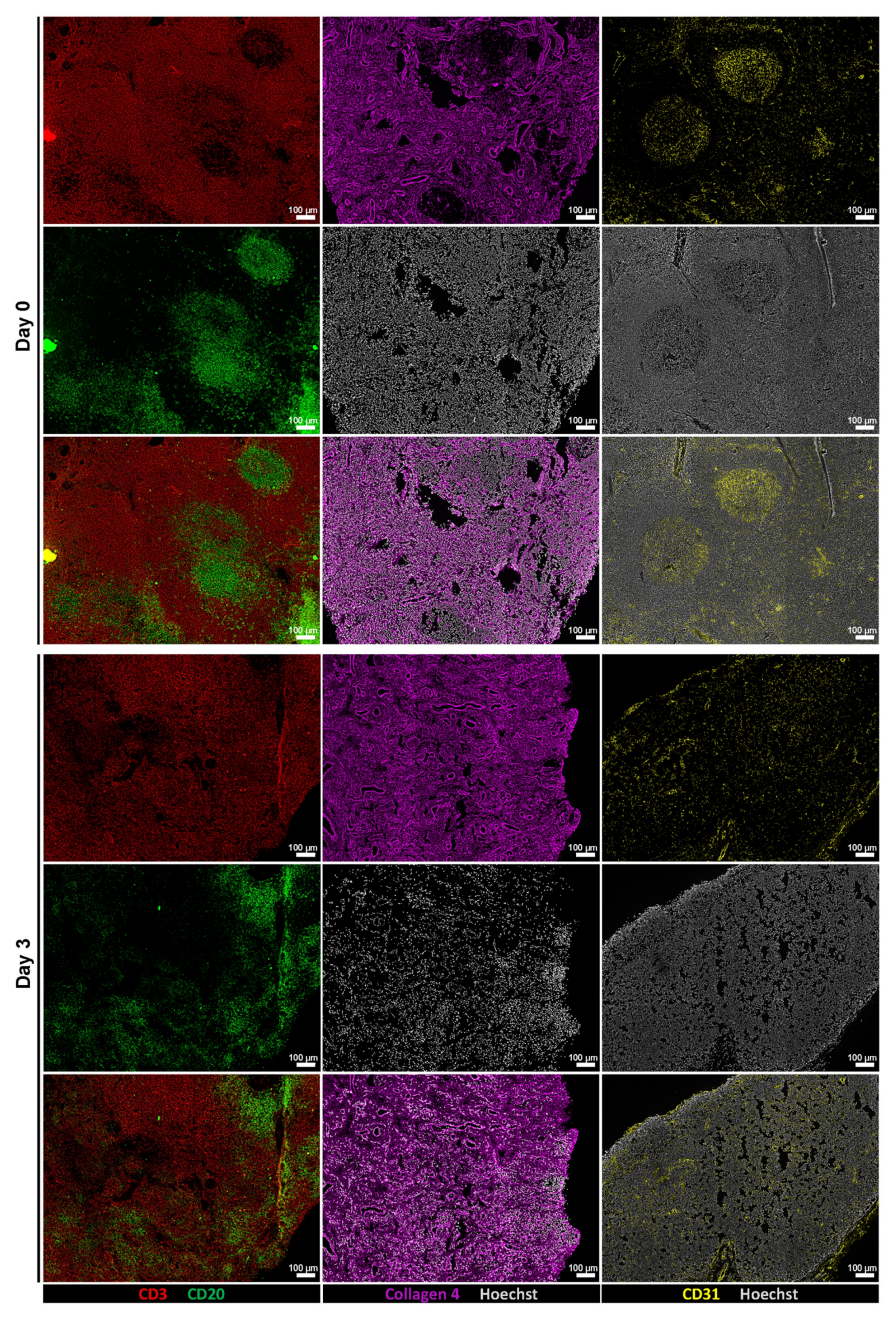
** Perfusion bioreactor culture preserves tissue architecture.** Representative low-magnification immunofluorescence images of tissue sections at Day 0 (top row) and after 3 days of *ex vivo* culture (bottom row). From left to right: CD3 (red) and CD20 (green) staining show preserved lymphoid compartmentalization; Collagen IV (magenta) indicates intact ECM; CD31 (yellow) highlights maintained vasculature. Scale bars: 100 μm.

To assess whether tissue architecture was preserved during *ex vivo* culture, we performed immunofluorescence staining and low-magnification imaging on tissue sections from Day 0 and Day 3 samples. Stainings for CD3 and CD20 revealed maintained lymphoid compartmentalization, while collagen IV and CD31 stainings demonstrated preserved extracellular matrix structure and vasculature, respectively (**Figure 3**). In addition, representative H&E staining showed that tissue morphology, including overall cellularity and structural organization, was largely maintained during the culture period (**Figure S6**). These findings indicate that key structural features of the tissue microenvironment remained intact throughout the culture period.

Taken together, our findings emphasize the robust performance of our bioreactor culture system in supporting the 3D culture of live, intact lymphoid tissues, establishing it as a promising platform for advanced modeling studies.

### Modeling ICIs treatment in lymphoma, metastatic cancer LNs, and benign LNs

**Figure 4:**
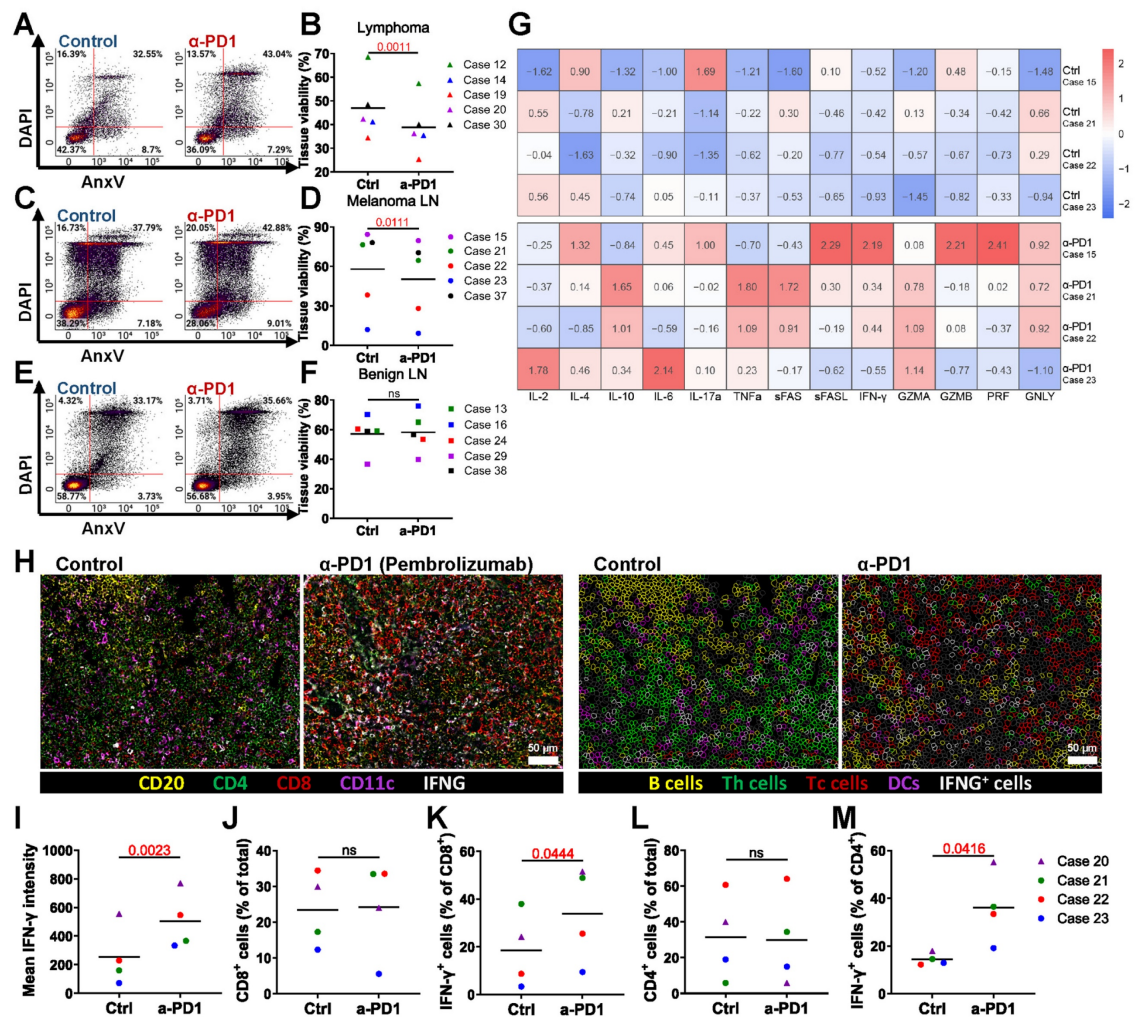
** Modeling ICI therapy.** Flow cytometry analysis after 3 days of pembrolizumab treatment shows: **(A-B)** Changes in lymphoma LN tissue (n = 5), **(C-D)** changes in melanoma metastasis LN tissue (n = 5), and **(E-F)** Immune cell changes in benign LN tissue (n = 5). **(G)** Heatmap representing relative soluble factor levels in culture media. **(H)** CODEX images showing expression of CD20, CD4, CD8, CD11c, and IFN-γ. Left: original overlay images with pseudocolor; right: cell annotation based on intensity thresholds (CD20: 1059; CD4: 2997; CD8: 5125; CD11c: 3532; IFN-γ: 4375). Scale bars: 50 μm. **(I)** Quantification of IFN-γ levels across samples by fluorescence intensity (n = 4). **(J)** Frequency of CD8^+^ Tc cells (n = 4). **(K)** Frequency of IFN-γ expressing Tc cells (n = 4). **(L)** Frequency of CD4^+^ Th cells (n = 4). **(M)** Frequency of IFN-γ expressing Th cells (n = 4). Statistics: paired t-test.

We tested the PD-1 inhibitor pembrolizumab in the bioreactor culture system to evaluate its effects on tissue viability. Flow cytometry data showed that a 3-day treatment with pembrolizumab led to a significant decrease in overall tissue viability of lymphoma tissue (**Figure 4A-B**) and LN tissue with melanoma metastasis (**Figure 4C-D**). In contrast, no significant difference in viability was observed in benign LN tissue with or without treatment (**Figure 4E-F**). These results suggest that pembrolizumab specifically affects malignant tissue without impacting benign tissue.

We were also interested in the changes in soluble factors in the culture medium elicited by anti-PD-1 treatment of bioreactors. Pembrolizumab-treated samples exhibited increased levels of IL-4, IL-6, IL-10, IL-17α, tumor necrosis factor-α (TNF-α), soluble (s)Fas, sFas-ligand, interferon-γ (IFN-γ), granzyme A (GZMA), GZMB, perforin (PRF), and granulysin (GNLY), while IL-2 levels remained comparable to the control. These findings indicate that pembrolizumab treatment promotes the release of cytokines associated with T cell activation in the bioreactor cancer treatment model (**Figure 4G**). Additionally, CODEX multiplexed staining confirmed the presence of immune cells and the expression of IFN-γ (**Figure 4H-I**). Although the frequency of CD8⁺ T cells was not increased (**Figure 4J**), the proportion of IFN-γ-expressing CD8⁺ T cells was elevated (**Figure 4K**). Similarly, CD4⁺ T cell frequencies remained stable, while IFN-γ⁺CD4⁺ T cells increased (**Figure 4L-M**).

Overall, these results indicate that our bioreactor culture system successfully models ICIs treatment and demonstrates that pembrolizumab reduces the viability of malignant LNs and enhances T cell-mediated immune responses.

### Modeling CAR T cell treatment

**Figure 5:**
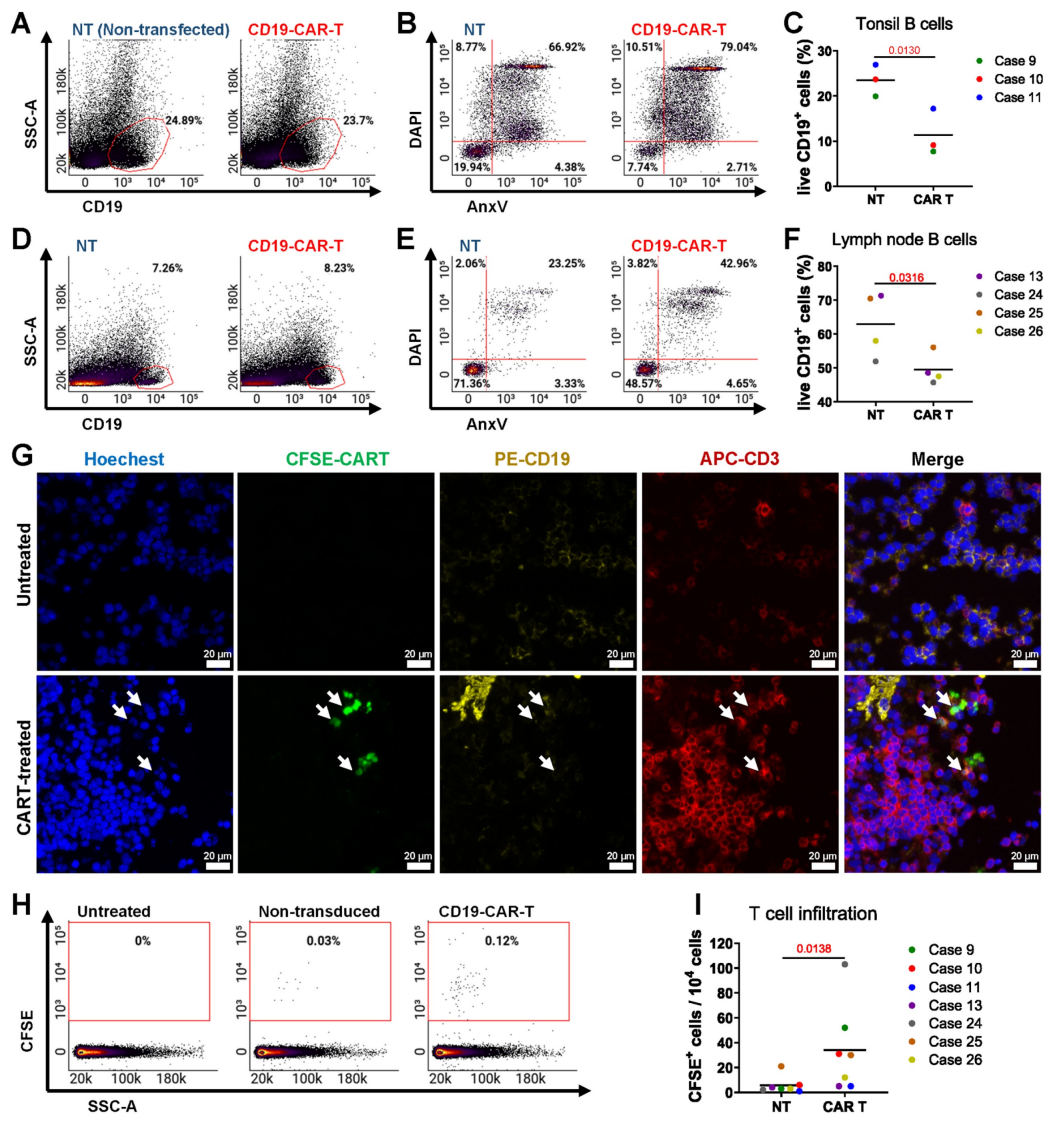
** Modeling anti-CD19 CAR T cell therapy in the bioreactor culture system.** Flow cytometry and immunofluorescence analyses of tonsil and LN tissues cultured with anti-CD19 CAR T cells or NT T cells for 3 days. **(A-B)** Representative flow cytometry plots showing the proportion and viability and **(C)** quantification of viable CD19^+^ B cells in tonsil tissue (n = 3). **(D-E)** Representative flow cytometry plots showing the proportion and viability of CD19^+^ B cells in LN tissue. **(F)** Quantification of viable CD19^+^ B cells in LN tissue (n = 4). **(G)** Representative image immunofluorescence staining showing the presence of CAR T cells in lymphoid tissue. Scale bars: 20 μm. **(H)** Representative flow cytometry plots comparing the presence of CAR T cells and NT T cells in tonsil and LN tissues. **(I)** Quantification of CAR T cells versus NT T cells (n = 7). Statistics: **(C, F)** paired t-tests; **(I)** ratio paired t-test.

We evaluated anti-CD19 CAR T cell therapy with tonsil and LN tissues, which are rich in CD19^+^ B cells. We introduced CAR T cells and non-transduced (NT) T cells into the bioreactors and maintained the treatment for 3 days. Post-culture flow cytometry analysis assessed CAR T cell efficacy in eliminating CD19⁺ B cells, with representative plots showing changes in their proportion and viability compared to NT T cell-treated controls. (**Figure 5A-B**). Quantification revealed a significant reduction in live CD19^+^ B cells in CAR T-treated tonsil tissues compared to NT controls (**Figure 5C**), and similar results were observed in LN tissues (**Figure 5D-F**). We confirmed CAR T cell infiltration in lymphoid tissues through IF staining (**Figure 5G**).

CAR T cells were more frequently found in the tissues compared to NT T cells, as evidenced by IF staining and quantification (**Figure 5H-I**). These data indicate that our bioreactor culture system effectively recapitulates the anti-CD19 CAR T cell activity of targeting and eliminating CD19^+^ B cells in lymphoid tissues. By enabling the evaluation of CAR T cell infiltration, cytotoxicity, and tissue-specific effects, this model provides a powerful tool for studying CAR T cell therapies in a controlled, physiologically relevant environment.

To evaluate whether the agarose coating used in bioreactor cultures affects CAR T cell behavior, we performed 2D co-culture experiments with or without a 0.5% agarose bottom layer. The presence of agarose did not significantly impact T cell viability or CAR T cell-mediated killing of B cell lymphoma target cell lines (**Figure S7A-B**), indicating compatibility with T cell function.

### Evaluating novel PI3K-enhanced CAR T cells in the 3D bioreactor culture system

**Figure 6:**
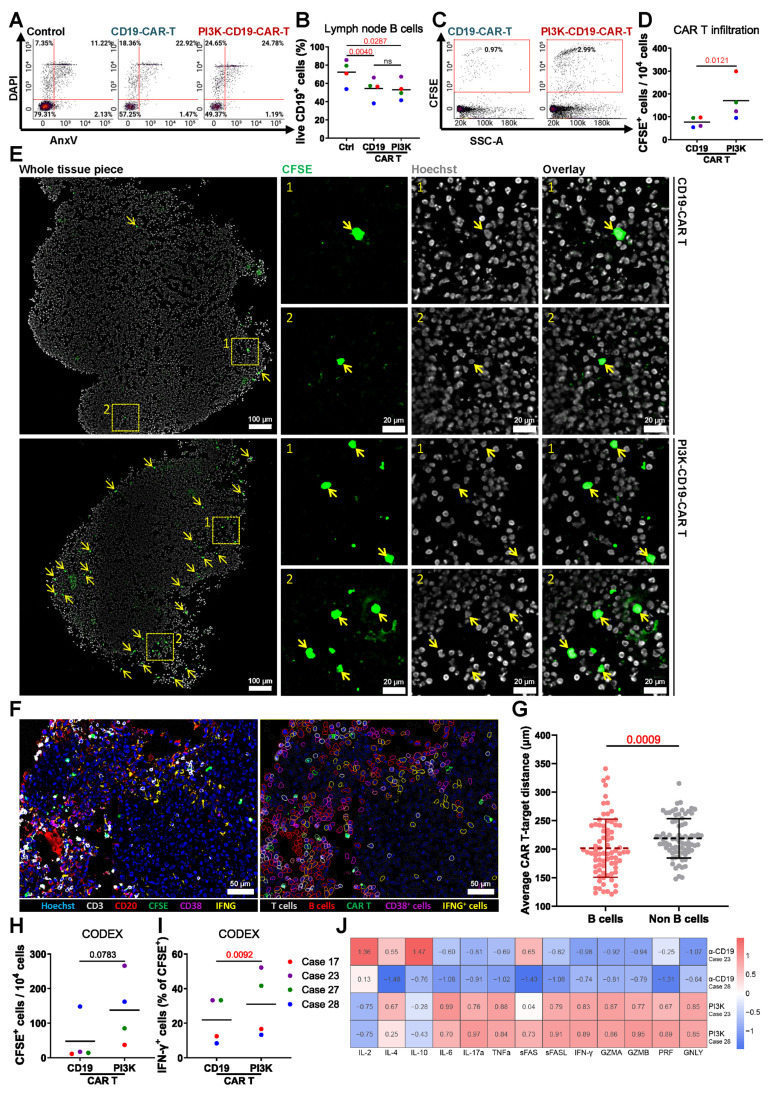
** Comparison of PI3K-enhanced CAR T Cells with conventional anti-CD19 CAR T cells. (A)** Representative flow cytometry plots and **(B)** quantification of viable CD19+ B cells in LN tissues following CD19-CAR T or PI3K-CAR T treatments (n = 4). **(C)** Representative flow cytometry plots and **(D)** quantification of CD19-CAR T and PI3K-CAR T cell infiltration into tissues. **(E)** Representative fluorescence images showing CD19-CAR T cell and PI3K-CAR T cell infiltration into LN tissues. CAR T cells are highlighted by yellow arrows and insets by rectangles. Scale bars: 100 μm (zoomed out), 20 μm (zoomed in). **(F)** A representative multiplex image and the corresponding segmented image illustrate the distribution of different cell types. Left: original overlay image with pseudocolor; Right: cell annotation based on intensity thresholds (CD3: 737; CD20: 632; CFSE: 1561; CD38: 375; IFN-γ: 357). Scale bar: 100 μm. **(G)** The average distances between each effector cell (CAR T cells) and target cells (B cells), as well as between each effector cell and non-target cells, were calculated. From 4 independent experiments: 9, 14, 30 and 24 effector cells; 307, 64, 264 and 243 target cells; and 812, 881, 1307 and 886 non-target cells were identified respectively, and used for distance analysis. **(H)** The abundance of CAR T cells in tissue. **(I)** percentage of IFN-γ expressing CAR T cells (n = 4). **(J)** Heatmap illustrating relative cytokine levels detected after CD19-CAR T or PI3K-CAR T treatments. Statistics: **(B)** one-way ANOVA with Tukey's post hoc test; **(D, G, H, I)** ratio paired t-tests.

Introducing specific point mutations in PIK3CD can enhance the functional properties of CAR T cells as demonstrated by Bucher et al. 34. We evaluated PI3K-19BBz CAR T cells with enhancement of the PI3K pathway by comparing them to conventional 19BB CAR T cells. Flow cytometry analysis showed no significant difference in the proportion of live CD19^+^ B cells between the two treatments (**Figure 6A-B**). However, greater numbers of PI3K-CAR T cells were found in tissues as analyzed by flow cytometry (**Figure 6C-D**) and fluorescence microscopy (**Figure 6E**).

To characterize the cellular composition and spatial organization of CAR T cells in treated tissues, we employed CODEX multiplexed fluorescence microscopy using CD3, CD20, CFSE, CD38, and IFN-γ. Spatial analysis was focused on PI3K-CAR T cell-treated tissues, where the abundance of CAR T cells was sufficient for robust evaluation (**Figure 6F**). Quantification of the average distances between PI3K-CAR T cells, B cells, and non-B cells revealed that PI3K-CAR T cells were significantly closer to B cells than to non-B cells, indicating selective targeting (**Figure 6G**). Additionally, CODEX analysis revealed higher numbers of PI3K-CAR T cells in LN tissues compared to conventional CAR T cells (**Figure 6H**), and a greater proportion of these cells expressed IFN-γ (**Figure 6I**).

Cytokine analysis of culture media from PI3K-CAR T cell-treated LN tissues revealed elevated levels of T cell activation-associated cytokines, including IL-4, IL-6, IL-17α, TNF-α, and IFN-γ, compared to tissues treated with control CAR T cells. Interestingly, PI3K-CAR T cell treatment also resulted in reduced levels of the immunosuppressive cytokine IL-10, although levels of the activation cytokine IL-2 were also decreased (**Figure 6J**). Collectively, these findings suggest that PI3K-CAR T cells elicit a more robust immune response than conventional CAR T cells.

In conclusion, PI3K-CAR T cells demonstrate enhanced tissue penetration and activation, suggesting that enhancement of the PI3K activity may improve CAR T cell therapeutic efficacy. Furthermore, our bioreactor culture system effectively models CAR T cell therapy, providing a valuable platform for developing and optimizing advanced CAR T cell products.

## Discussion

Over the past decade, cancer immunotherapies have gained significant attention due to their efficacy in treating refractory cancers and delivering durable therapeutic outcomes 49. The TME has been recognized as a critical factor influencing immune reaction, treatment responsiveness and clinical outcomes 12. Consequently, due to the heterogeneity of both the tumor and its microenvironment, a key challenge remains in determining which patients will benefit from immunotherapies and how to effectively personalize these strategies. This is especially crucial for managing late-stage lymphoma and metastatic cancers, where the spread of chemotherapy-resistant neoplastic cells is a leading cause of cancer-related mortality. In such cases, conventional therapies are often no longer viable, making alternative approaches like immunotherapy a preferable option 50.

Functional precision medicine (FPM) combines direct *ex vivo* drug testing on patient-derived tumor samples with genomic and clinical information to guide personalized therapy choices. Unlike purely molecular profiling, which infers drug sensitivity from genetic or transcriptomic signatures, FPM measures the actual functional response of living tumor cells to therapeutic agents 51. This approach has shown clinical promise in hematological malignancies, where rapid turnaround is possible, and has been linked to improved patient outcomes in several prospective studies 52,53.

Our lymphoid bioreactor extends the scope of FPM by enabling functional testing of immunotherapies in a preserved immune TME. By maintaining the structural and cellular complexity of lymphoid tissues, this system allows assessment of both direct anti-tumor effects and immune-mediated responses. Such a platform could complement current FPM workflows by providing actionable, patient-specific data for immunotherapeutic decision-making, particularly in lymphomas where standard drug-sensitivity assays fail to capture immune-tumor interactions.

Traditional 2D cell line models lack the complexity of the 3D TME structure, which includes immune cells, vasculature, fibroblasts, and ECM 54. While 3D models like spheroids and organoids improve structural relevance, they still fall short in replicating full tissue heterogeneity 22,23. *In vivo* models such as patient-derived xenografts (PDXs) maintain tumor architecture but lack a human immune system, limiting their use for immunotherapy studies 55. However, PDXs are also costly and low-throughput. These limitations underscore the need for more physiologically relevant and scalable models in cancer research 56.

Short-term (3-day) bioreactor cultures with patient-derived tissues enable rapid assessment of treatment efficacy, supporting personalized therapy decisions. Clinically, small surgical samples could be cultured in parallel with pathology to guide therapeutic selection. Embedding lymphoid tissue in 0.5% agarose, rather than collagen sponges or static plates, preserved 3D structure, maintained medium exchange, and preserved immune, stromal, and tumor cell viability comparable to fresh tissue 46,57. To further extend T and B cell survival, cytokine supplementation beyond IL-2 and IL-7, such as BAFF or IL-15, may be explored 58,59.

The perfusion system's dynamic medium flow supports tissue viability by delivering nutrients and oxygen while removing waste, mitigating necrosis often seen in static cultures 60. This continuous circulation better mimics physiological fluid dynamics, which are known to influence cell survival, differentiation, and immune-tumor interactions. Such conditions likely contribute to the sustained viability of both immune and malignant cells in our model, making it more representative of the *in vivo* microenvironment than traditional 2D or static 3D cultures.

Compared to the 3D perfusion bioreactor model of Bonaiti et al., which used human tonsil explants to study vaccine responses 61, our platform employs LNs, which are more representative of tumor immunobiology. This system maintained high viability over three days, providing a relevant window for *ex vivo* drug screening. Using this approach, we demonstrated its ability to model immune checkpoint blockade and CAR T cell therapy, revealing differences in efficacy and immune modulation. These findings position the perfusion bioreactor as a versatile tool for comparative immunotherapy evaluation in a physiologically relevant, controlled setting. We selected a 3-day culture period as the standard endpoint, as tissue viability remained high and comparable to fresh lymph nodes at this time point, while a downward trend was observed by day 5. Additionally, previous studies support 3-day immune checkpoint inhibitor and 2-day CAR T cell treatments as effective windows for drug screening assays 62,63.

Our findings highlight that the 3D perfusion bioreactor preserves the immune TME's architecture while allowing detection of dynamic immune responses to therapy. This structural integrity enables spatial mapping of cell-cell interactions 41, such as T cell infiltration and proximity to target cells, which are often lost in conventional cultures. Such capabilities make the model valuable for elucidating mechanisms of immune-based therapies and for bridging the gap between static *ex vivo* assays and *in vivo* studies.

In conclusion, our perfusion bioreactor provides a robust platform for short-term culture of human lymphoid tissues, preserving their viability, architecture, and immune microenvironment. By enabling functional testing of immunotherapies such as PD-1 inhibitors and CAR T cells, this system offers a valuable tool for preclinical drug screening and holds promise for advancing personalized cancer immunotherapy.

## Limitations

While the bioreactor system offers a valuable platform for *ex vivo* modeling of the human TME, several biological, technical, and logistical limitations must be acknowledged.

Biological and technical constraints: The approach relies on fresh surgical specimens, making tissue availability and quality highly variable. Delays between resection and processing can reduce tissue viability and affect experimental outcomes. The embedding of tissue in 0.5% agarose, although beneficial for maintaining 3D structure and facilitating tissue viability, may introduce altered cell behavior, which could influence immune cell function and drug responses. Additionally, viability analyses were conducted on mechanically dissociated single-cell suspensions using Annexin V/DAPI flow cytometry. This method, while enabling multiplexed apoptosis and necrosis measurements across immune subsets, may bias viability results due to differential sensitivity of fragile or apoptotic cells to dissociation. Complementary *in situ* assays such as cleaved caspase-3 or TUNEL staining could preserve spatial context and provide more nuanced viability information in future work.

Sampling and statistical limitations: Image-based analyses were restricted to a limited number of explants and tissue sections, potentially limiting the generalizability of spatial findings due to inter-explant variability. Although lymphomas generally exhibit lower intratumor heterogeneity than solid tumors 64, spatial variations in immune cell distribution and tumor architecture may still influence treatment responses. The lack of multiregional sampling further constrains the ability to fully capture this heterogeneity. Donor-to-donor variability also presents a challenge, as patient-specific differences in immune composition, tumor characteristics, and prior treatments may impact experimental reproducibility and interpretation. In addition, cytokine profiling was performed on few samples, which limits statistical robustness and necessitates cautious interpretation of mechanistic claims. Finally, the relatively small sample sizes and subset analyses reduce statistical power, and future studies should incorporate larger, more diverse cohorts to strengthen conclusions.

Immunophenotyping scope: While T cell subsets (CD4⁺ and CD8⁺) were analyzed, other important immune populations such as regulatory T cells (Tregs, e.g., FOXP3⁺), and detailed B cell subtypes (naïve, memory, germinal center, plasma cells) were not comprehensively characterized. This limits the understanding of the full immune landscape and its modulation by immunotherapies in the lymphoid TME.

Throughput and practical limitations: The platform currently supports a low throughput, limiting the number of drug conditions that can be tested per tissue sample. Combined with inherent tissue heterogeneity, this restricts the breadth and scalability of experiments. Future improvements to increase throughput, standardize sample processing, and enable biobanking of viable tissue will enhance mechanistic and retrospective analyses.

## Supplementary Material

Supplementary figures and tables.

Supplementary material: cell distance.

## Abbreviations

CAR: chimeric antigen receptor
TME: tumor microenvironment, LN, lymph node
ICIs: immune checkpoint inhibitors
ECM: extracellular matrix
2D: two-dimensional
3D: three-dimensional
NT: non-transduced
IF: immunofluorescence
H&E: hematoxylin and eosin
BME: basement membrane extract
IL: interleukin
COI: cells of interest
NK: natural killer
WBCs: white blood cells
TNF-α: tumor necrosis factor-α
IFN-γ: interferon-γ
GZMA: granzyme A
PRF: perforin
GNLY: granulysin
FPM: functional precision medicine
Tregs: regulatory T cells
