# Supplementary material for: Modeling immunotherapies in live 3D human cancer tissue bioreactors
Source: Theranostics. 2026 Jan 14;16(8):3928–45. doi: 10.7150/thno.118298 (PMC12905668; doi:10.7150/thno.118298)
Supplement: Supplementary file 1 — Supplementary figures and tables. [file thnov16p3928s1.pdf]

SUPPLEMENTARY FIGURES AND LEGENDS

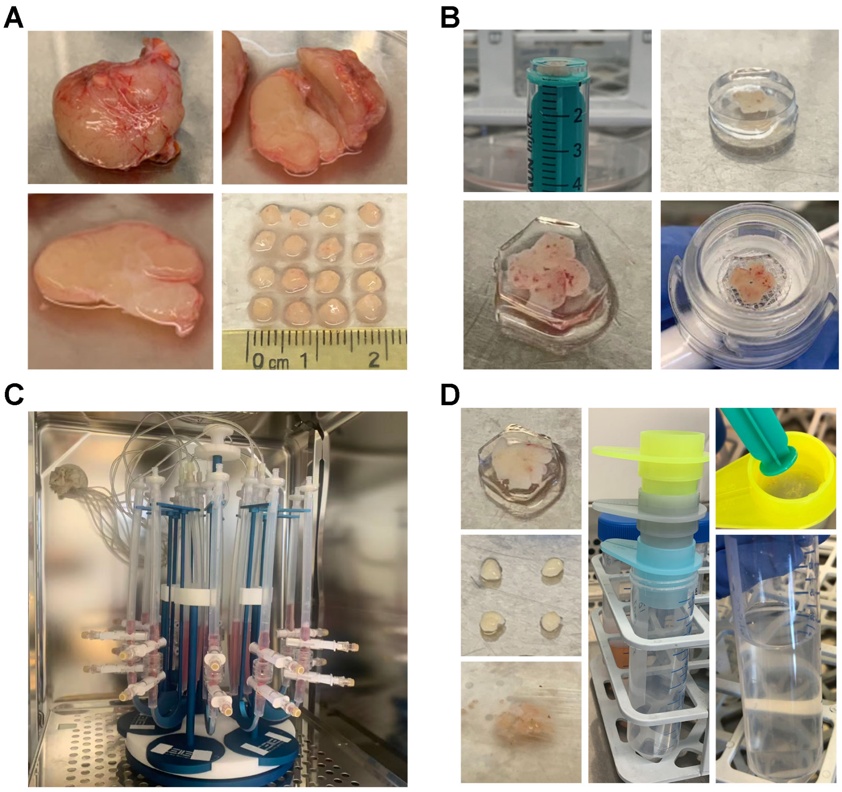

Figure S1. Main steps of tissue preparation and bioreactor culture for human lymphoid tissues

(**A**) Cutting lymphoid tissue into 3×3×3 mm pieces with a scalpel. (**B**) Embedding tissue pieces in 0.5% agarose. (**C**) Culturing embedded tissue in the 3D perfusion bioreactor inside an incubator. (**D**) Harvesting tissue samples after culture for downstream analysis.

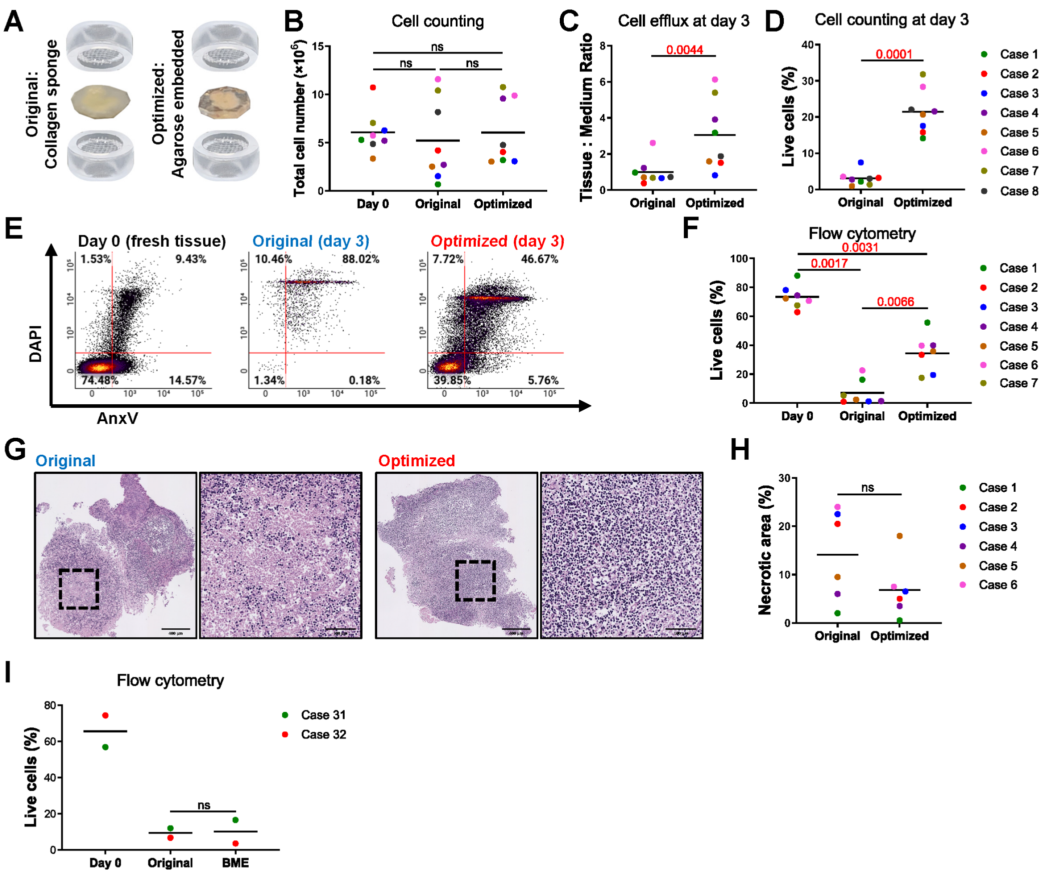

Figure S2. Improved cellular retention and viability in tonsil tissue cultures using optimized agarose embedding

(**A**) Schematic comparing the original collagen sponge setup with the optimized agarose embedding setup. (**B**) Estimated total cell numbers in each bioreactor before and after culture based on cell counting (n = 8). (**C**) Cell count analysis shows reduced efflux of cells into the medium in agarose-embedded tissues (n = 8). (**D**) Trypan blue staining confirms higher tissue viability in agarose-embedded samples (n = 8). (**E**) Representative DAPI/Annexin V flow plots for cell viability. **(F**) flow cytometry analysis demonstrates improved retention of viable cells in agarose-embedded tissues (n = 7). (**G**) H&E staining highlights necrotic areas in tissue sections. Scale bars: 400 µm (zoomed out), 100 µm (zoomed in). (**H**) Necrotic tissue area of post-culture tissue with or without agarose embedding (n = 6). (**I**) Evaluation of BME as an alternative ECM matrix for tissue embedding (n = 2). Statistics: (B, I) Friedman test with Dunn's post hoc test; (C, D) paired t-test; (F) Lognormal RM one-way ANOVA with Tukey's post hoc test; (H) Wilcoxon test.

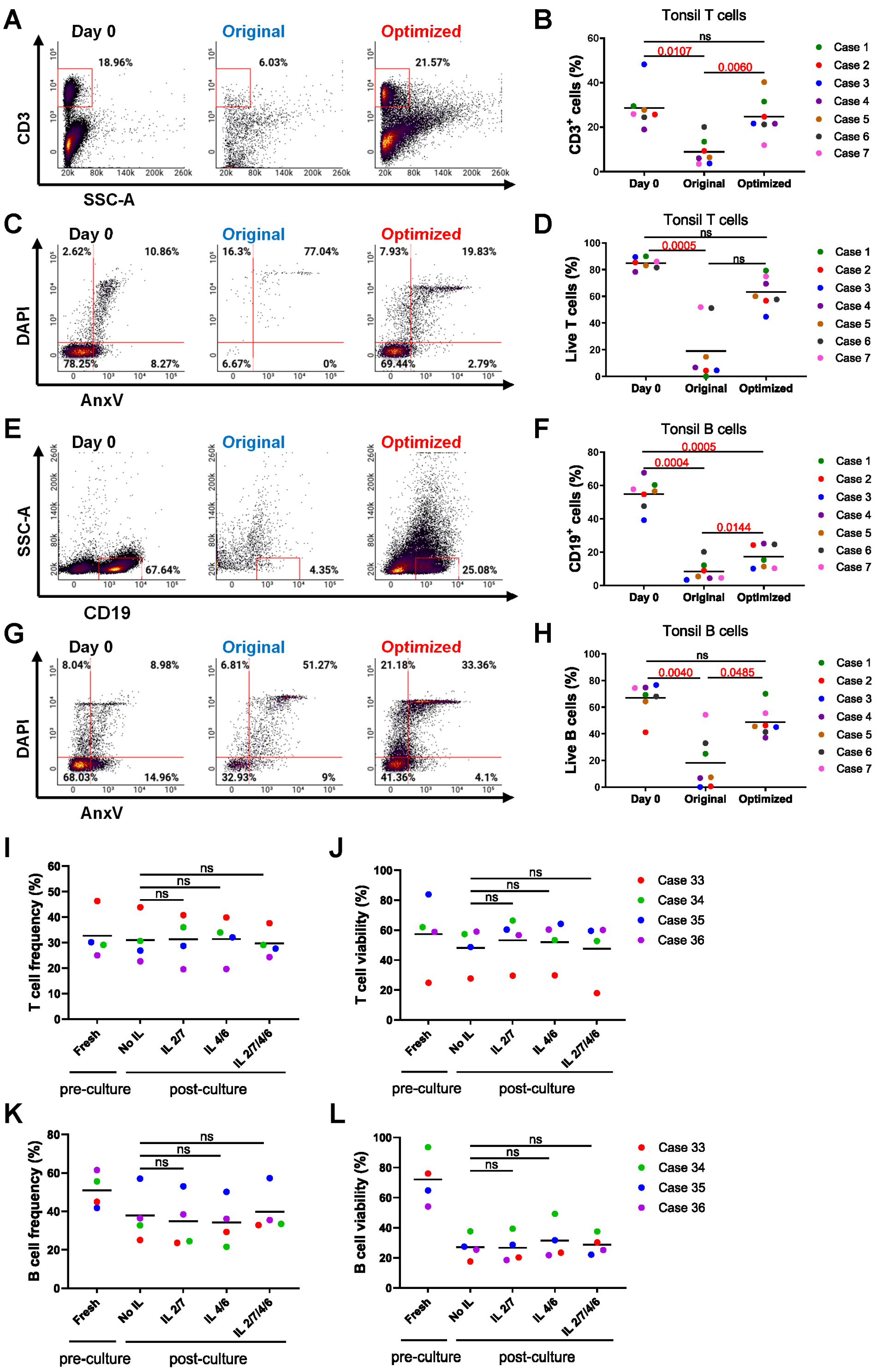

Figure S3. Preservation of immune cell subsets in tonsil tissue cultures using agarose embedding

(**A**) Representative flow cytometry plots showing $CD3^{+}$ T cells. (**B**) Quantitative analysis shows T cell frequency. (**C**) Representative plots of DAPI/Annexin V staining to assess T cell viability. (**D**) Quantification shows T cells viability. (**E**) Representative flow cytometry plots showing $CD19^{+}$ B cells. (**F**) Quantitative analysis of B cells

frequency. (**G**) Representative plots of DAPI/Annexin V staining to evaluate B cell viability. (**H**) Quantitative analysis of B cell viability (n = 7). (**I–J**) Flow cytometry analysis of $CD3^+$ T cells frequency and viability in human tonsil tissue cultured with different cytokine combinations for 3 days in perfusion bioreactors. (**K–L**) Flow cytometry analysis of $CD19^+$ B cells under the same conditions. Statistics: (B, F) Lognormal RM one-way ANOVA with Tukey's post hoc test. (I, L) RM one-way ANOVA with Tukey's post hoc test. (D, H, J, K) Friedman test with Dunn's post hoc test.

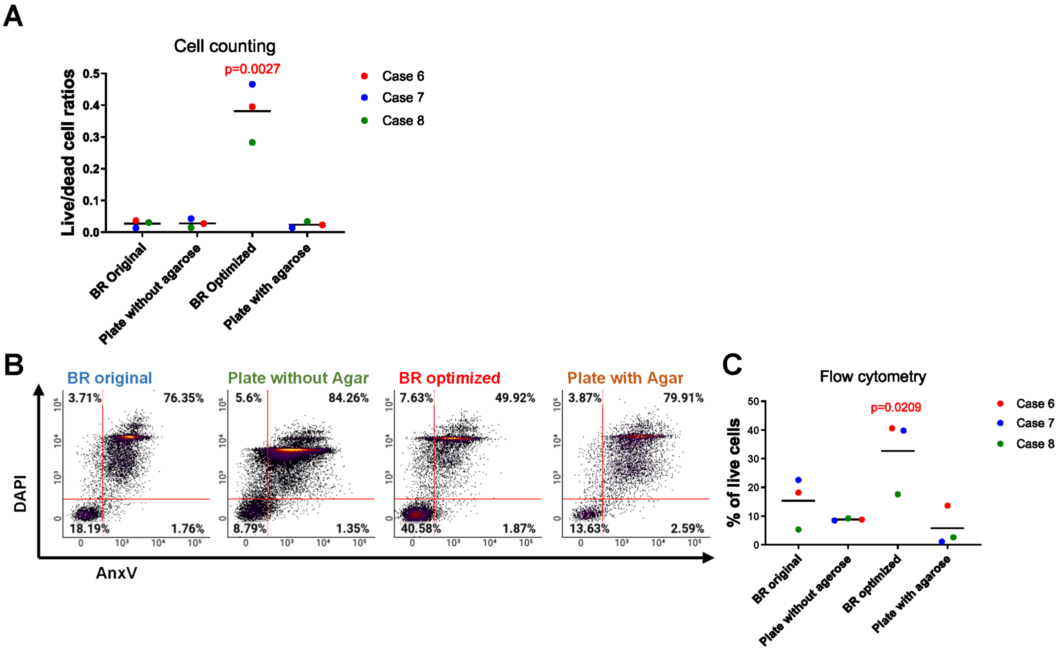

Figure S4. Comparison of static plate culture and bioreactor setups for tonsil tissue culture.

(**A**) Cell counting analysis and (**B-C**) flow cytometry analysis comparing bioreactor and static plate culture setups with or without agarose embedding (n = 3). Statistics: RM one-way ANOVA.

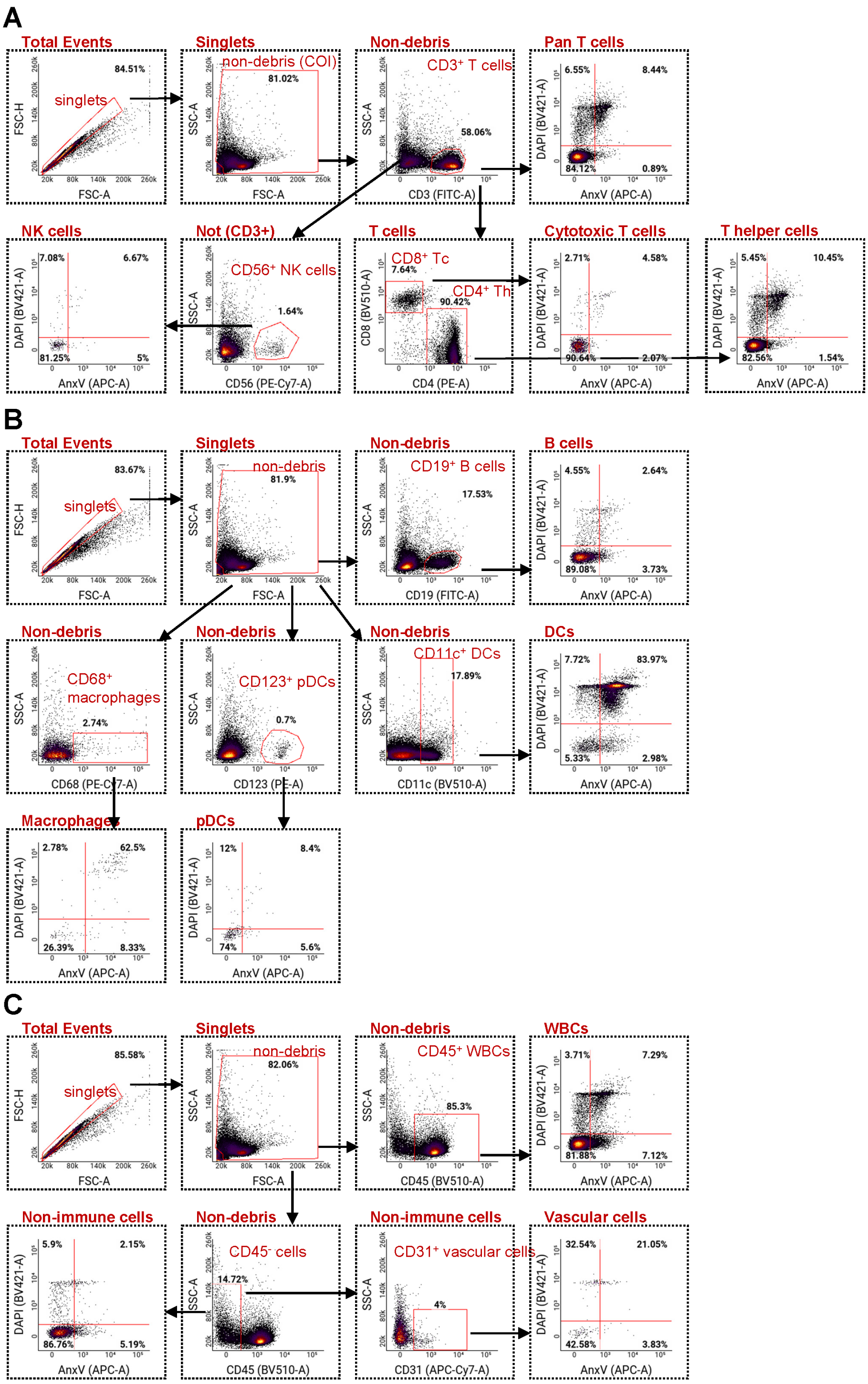

Figure S5. Flow cytometry gating strategy for immune cell subsets in panel 1-3. (**A**) Doublets and cell debris were first excluded. Gating strategy was used to identify CD3$^+$ T cells, CD4$^+$ T helper cells, CD8$^+$ cytotoxic T cells, and CD3$^-$CD56$^+$ natural killer (NK) cells. (**B**) Gating strategy for CD19$^+$ B cells, CD68$^+$ macrophages, CD123$^+$ plasmacytoid dendritic cells (pDCs), and CD11c$^+$ conventional dendritic cells (DCs). (**C**) Gating strategy for CD45$^+$ WBCs and CD45- non-immune cells.

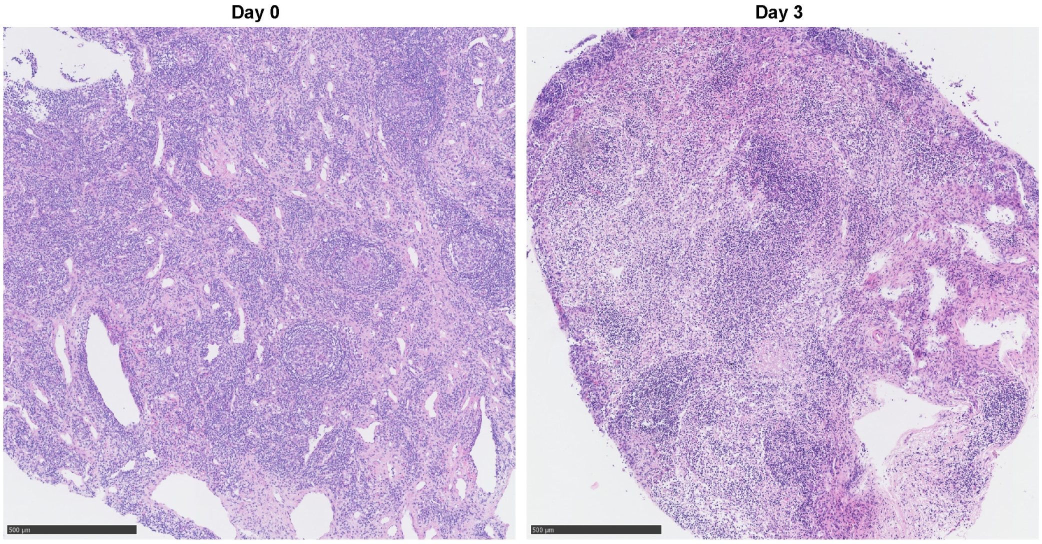

Figure S6. Tissue morphology before and after *ex vivo* culture.

(A) Representative H&E staining of tissue sections from Day 0 fresh tissue and Day 3 post-culture tissue samples.

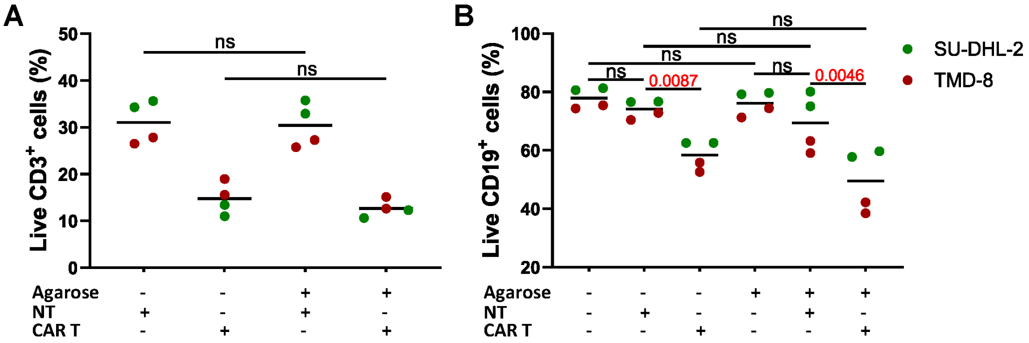

Figure S7. Evaluation of agarose coating effects on CAR T cell viability and function.

(A) Viability of NT cells and anti-CD19 CAR T cells after 48-hour culture in wells with or without 150 µL of 0.5% agarose coating. (B) Cytotoxic activity of CAR T cells against SU-DHL-2 and TMD-8 DLBCL cell lines in the presence or absence of agarose (n = 4, including 2 technical replicates and 2 biological replicates). Statistics: RM one-way ANOVA with Tukey's post hoc test.

TABLES

Table S1. Key resources

RESOURCE	SOURCE	IDENTIFIER
Fluorophore-labeled antibodies		
APC anti-human CD3	BioLegend	300311
FITC anti-human CD3	BioLegend	300306
PE anti-human CD4	BioLegend	317410
BV510 anti-human CD8	BioLegend	344732
PE anti-human CD19	BioLegend	302208
FITC anti-human CD19	BioLegend	302206
PE-Cy7 anti-human CD56	BioLegend	362509
BV510 anti-human CD11c	BioLegend	301633
PE anti-human CD123	BioLegend	306006
PE-Cy7 anti-human CD68	BioLegend	333815
BV510 anti-human CD45	BioLegend	368525
APC-Cy7 anti-human CD31	BioLegend	303119
APC anti-mouse IgG	BioLegend	405308
PE anti-rabbit IgG	BioLegend	406421
Antibodies for CODEX or IF		
anti-CD3 (Oligo 20)	BioLegend	300402
anti-CD4 (Oligo 28)	BioLegend	357402
anti-CD5 (Oligo 75)	BioLegend	300602
anti-CD8 (Oligo 43)	BioLegend	344702
anti-CD11c (Oligo 44)	BD Biosciences	555391
anti-CD19 (Oligo 2)	BioLegend	302202
anti-CD20 (Oligo 48)	Novus	NBP2-54591
anti-CD31 (Oligo 68)	BioLegend	303102
anti-CD38 (Oligo 66)	BioLegend	356602
anti-Collagen 4 (Oligo 33)	Abcam	ab6586
anti-IFN-γ (Oligo 30)	Akoya biosciences	240081
anti-FITC (Oligo 6)	BioLegend	408301
Chemicals and biological reagents		
Pembrolizumab	Selleckchem	A2005
EDTA	ROTH	X986.2
DAPI	BioLegend	422801
APC-Annexin V	BioLegend	640920
DMEM / F12 with 15mM HEPES	ROTH	9031.1
Penicillin-Streptomycin	VWR-Biowest	L0022-100
Amphotericin B	ThermoFisher	15290026
Nicotinamide	Sigma-Aldrich	N0636
NAC	Sigma-Aldrich	A7250
Human serum	Sigma-Aldrich	H5422
Interleukin-2	Miltenyi Biotec	130-097-742

Interleukin-7	Miltenyi Biotec	130-093-937
Interleukin-15	Miltenyi Biotec	130-095-760
pCMV-AncBE4max	Addgene	112095
AgeI-HF	NEB	R3552
T7 MEGAscript Kit	ThermoFisher	AMB13345
CleanCap AG	Tebubio	N-7113
PolyA-tailed	ThermoFisher	AM1350
sgRNA	IDT	N/A
IDTE pH7.5 buffer	IDT	11-05-01-05
NaCl	VWR Chemicals	27810.295
KCl	Merck	7447-40-7
$MgCl_2$	ROTH	KK36.1
$CaCl_2$	ROTH	CN92.1
HEPES	ROTH	HN77.3
CellTrace™ CFSE dye	ThermoFisher	C34554
Bovine serum albumin	BioSell	BSA.PF.0100
CFSE	ThermoFisher	C34554
Agarose	Biozym	840004
LEGENDplex™ Human CD8/NK Panel (13-plex)	BioLegend	741186
Fetal Calf Serum (FCS)	Sigma-Aldrich	F7524
PBS 10x	Sigma-Aldrich	D1408
DMSO	ROTH	7029.4
Trypan blue	Sigma-Aldrich	T8154
Triton X-100	ROTH	3051.3
Na_2HPO_4	Sigma-Aldrich	S7907-500G
NaH_2PO_4	Sigma-Aldrich	S9638-250G
Tris	ROTH	4855.2
BS^3	ThermoFisher	21580
mouse IgG	Biozol	JIM-015-000-003
rat IgG	Biozol	JIM-012-000-003
Sheared salmon sperm DNA	ThermoFisher	AM9680
Non-fluorescent oligonucleotides mix	Biomers	N/A
Poly-L-Lysine	Sigma-Aldrich	P8920-100ML
Acetone	neoFroxx	LC-4916.2
Methanol	Honeywell	32213
IDTE pH7.5	IDT	11-05-01-05
Hematoxylin	Sigma-Aldrich	1.09253
Eosin	Sigma-Aldrich	318906
Isopropanol	SAV Liquid Production	67-63-0
Ethanol	SAV Liquid Production	64-17-5
PFA 16%	ThermoFisher	0433368.9M
X-Vivo15 medium	Lonza	BEBP02-061Q
Cultrex BME	R&D	3432-005-01
Equipment and Consumables		

Clean bench	Heraeus	HB2448K
Scalpel	Pfm medical	02.001.30.021
Petri dish	Greiner Bio-One	664160
Tissue-Tek Cryomold	Sakura	SA 62534-25
Tissue-Tek O.C.T. compound	Sakura	SA62550
Syringe 5 ml	B. Braun	4606051V
Falcon tube 50 ml	Cellstar	227261
Cell strainer 100 µm	pluriSelect	45-57100-51
Cell strainer 70 µm	pluriSelect	43-57070-51
Cell strainer 40 µm	pluriSelect	43-57040-51
20G needle	B. Braun	4657519
Centrifuge	Thermo Heraeus	Multifuge 1S-R
Incubator	Heracell	51013568
U-CUP bioreactor	Cellec Biotek	N/A
U-CUP rack	Cellec Biotek	N/A
Syringe pump	Harvard Apparatus	MA1 70-3xxx
Silicone adaptors Ø10mm x 4 mm	Cellec Biotek	URD10H04
Nylon meshes Ø10mm	Cellec Biotek	N/A
Collagen sponges	Cellec Biotek	N/A
Polystyrene tubes	Falcon	352058
Neubauer chamber	Hecht Assistent	N/A
Cytoseal	Epredia	8312-4
Coverslips 24 x 50 mm	Marienfeld Superior	0101222
Coverslips 22 x 22 mm	Marienfeld Superior	0101052
BONDIC pen	Bondic VIKO UG	SW10014
TOMO adhesion slides	Matsunami Glass	TOM-1190
LED Pads	Aibecy	A5 25 '000 Lux
Cryostat	Leica	CM3050S
All-in-one fluorescence microscope	Keyence	BZ-X810
LSR II Flow Cytometer	BD Biosciences	N/A
FACS Canto II	BD Biosciences	N/A
PhenoCycler (CODEX®)	Akoya Biosciences	N/A
Neon Transfection System	Thermo Fisher	N/A
24-well plates	Falcon	3003005
6-well plates	Falcon	3003055
Biological material		
Human lymphoid tissue	University Hospital Tübingen	
anti-CD19 CAR T cells	Düsseldorf University, Prof. Sascha Dietrich’s Lab	
PI3K-CD19 CAR T & Control CAR T cells	Tübingen University, Prof. Judith Feucht’s Lab	
SU-DHL-2 & TMD-8 cell lines	Tübingen University, Prof. Alexander Weber’s Lab	

Table S2. Composition of buffers, working solutions and culture medium

70% Ethanol		
Reagent	Final concentration	Amount
100% Ethanol		700 ml
ddH_2O		300 ml
Total	70%(v/v)	≈ 1000 ml
Store at RT		

1 x PBS		
Reagent	Final concentration	Amount
10 x PBS		100 ml
ddH_2O		900 ml
Total	1 x	1000 ml
Autoclave sterilization, store at RT		

EDTA-PBS		
Reagent	Final concentration	Amount
1 x PBS		1000 ml
EDTA	2 mM	744.5 mg
Total		≈ 1000 ml
Autoclave sterilization, store at RT		

PBS with 0.1% BSA		
Reagent	Final concentration	Amount
1 x PBS		10 ml
BSA		0.01 g
Total	0.1%	≈ 10 ml
Store at -20°C		

IL-2 aliquots		
Reagent	Final concentration	Amount
PBS with 0.1% BSA		1700 µl
IL-2 (1.7 x 10^7U/mg)		10 µg
Total		≈ 1700 µl
Aliquots	2000U / 20µl	20 µl x 85 vials
Store at -80°C		

IL-7 aliquots		
Reagent	Final concentration	Amount
PBS with 0.1% BSA		400 µl

IL-7 (2 x 10^7U/mg)		10 µg
Total		≈ 400 µl
Aliquots	10000U / 20µl	20 µl x 20 vials
Store at -80°C		

82

20 mM N-Acetylcysteine (NAC)		
Reagent	Final concentration	Amount
NAC		48.96 mg
ddH_2O		15 ml
Total	20 mM	≈ 15 ml
Store at -20°C		

83

20 mM Nicotinamide		
Reagent	Final concentration	Amount
Nicotinamide		36.63 mg
ddH_2O		15 ml
Total	20 mM	≈ 15 ml
Store at -20°C		

84

Culture medium aliquots		
Reagent	Final concentration	Amount
DMEM / F12 with 15mM HEPES		78 ml
Penicillin-Streptomycin	1 %	1 ml
amphotericin B	1 %	1 ml
20 mM NAC	1 mM	5 ml
20 mM Nicotinamide	1 mM	5 ml
Human serum	10 %	10 ml
Total		100 ml
Aliquots		10 ml x 10 tubes
Store at 4°C		

85

Final culture medium (used for bioreactors and static culture)		
Reagent	Final concentration	Amount
Culture medium aliquot		10 ml
IL-2 aliquots	200 U/ml	20 µl
IL-7 aliquots	1000 U/ml	20 µl
Total		≈ 10 ml
Freshly add ILs before use, every bioreactor needs 10 ml final culture medium		

86

0.5% Agarose

Reagent	Final concentration	Amount
Agarose		0.1 g
PBS		20 ml
Total	0.5%	≈ 20 ml
Autoclave sterilization or boiling for 1h, store at RT		

CFSE staining reagent		
Reagent	Final concentration	Amount
CellTrace™ CFSE dye		1 vial
DMSO		18 µl
Total	5 mM	≈ 18 ml
Store at -20°C		

Annexin V binding buffer		
Reagent	Final concentration	Amount
HEPES	10 mM	2.38 g
NACL	150 mM	8.77 g
KCL	5 mM	372.75 mg
$MgCl_2$	1 mM	95.21 mg
$CaCl_2$	1.8 mM	199.76 mg
ddH_2O		To 1000 ml
Total		1000 ml
Store at RT		

S1 buffer		
Reagent	Final concentration	Amount
EDTA	5 mM	1.46 g
10 x PBS	1 x	100 ml
BSA	5%	5 g
ddH_2O		900 ml
Total		≈ 1000 ml
Store at 4°C		

S2 buffer		
Reagent	Final concentration	Amount
1 x S1		500 ml
Na_2HPO_4	61 mM	8.66 g
NaH_2PO_4	39 mM	4.68
5 M NaCl	250 mM	50 ml
ddH_2O		450 ml
Total		≈ 1000 ml

Store at 4°C		

S4 buffer		
Reagent	Final concentration	Amount
1 x S1	0.9 x	900 ml
5 M NaCl	0.5 M	100 ml
Total		1000 ml
Store at 4°C		

H2 buffer (reaction buffer of codex multicycle)		
Reagent	Final concentration	Amount
1 M Tris pH= 7.5	0.1 M	10 ml
Triton X-100	0.1% (w/v)	1 ml
$MgCl_2$	10 mM	0.95 g
5 M NaCl	150 mM	30 ml
ddH_2O		960 ml
Total		≈ 1000 ml
Store at RT		

Bleaching solution		
Reagent	Final concentration	Amount
1 x PBS		20 ml
30% H_2O_2		3.6 ml
1M NaOH		0.64 ml
Freshly make before use		

CODEX blocking solution (also diluent for antibodies cocktail)		
Reagent	Final concentration	Amount
1 x S2		234 µl
mouse IgG	65 µg/ml	15 µl
rat IgG	65 µg/ml	15 µl
Sheared salmon sperm DNA	43 µg/ml	15 µl
Non-fluorescent oligonucleotides mix	0.5 mM	21 µl
Total		300 µl
Freshly make before use		

Table S3. Patient samples and diagnosis

Case #	Tissue type	Pathological diagnosis	Disease Status and Prior Treatment
Case 1	adenoids	adenoid hypertrophy	benign
Case 2	adenoids	adenoid hypertrophy	benign
Case 3	tonsil	chronic tonsillitis	benign
Case 4	adenoids	adenoid hypertrophy	benign
Case 5	tonsil	chronic tonsillitis	benign
Case 6	tonsil	chronic tonsillitis	benign
Case 7	tonsil	chronic tonsillitis	benign
Case 8	tonsil	chronic tonsillitis	benign
Case 9	tonsil	chronic tonsillitis	benign
Case 10	tonsil	chronic tonsillitis	benign
Case 11	tonsil	chronic tonsillitis	benign
Case 12	lymph node	T cell lymphoma	primary disease with no prior treatment
Case 13	lymph node	chronic lymphadenitis	benign
Case 14	lymph node	classical Hodgkin lymphoma	primary disease with no prior treatment
Case 15	lymph node	melanoma metastasis	relapsed disease after surgery
Case 16	lymph node	reactively changed lymph nodes	benign
Case 17	lymph node	melanoma metastasis	primary disease with no prior treatment
Case 18	lymph node	nonspecific lymphadenopathy	benign
Case 19	lymph node	diffuse large B-cell lymphoma	relapse after CHOP and stem cell transplant
Case 20	lymph node	classical Hodgkin lymphoma	primary disease with no prior treatment
Case 21	lymph node	melanoma metastasis	primary, prior pembrolizumab treatment
Case 22	lymph node	melanoma metastasis	primary disease with no prior treatment
Case 23	lymph node	melanoma metastasis	relapse, treated with nivolumab
Case 24	lymph node	Tumor-free lymph node	benign
Case 25	lymph node	chronic lymphadenitis	benign
Case 26	lymph node	follicular lymphoma	relapse, treated with obinutuzumab and CHOP
Case 27	lymph node	reactive plasmacytosis	benign
Case 28	lymph node	classical Hodgkin lymphoma	primary disease with no prior treatment
Case 29	lymph node	dermatopathic lymphadenitis	benign
Case 30	lymph node	follicular center lymphoma	primary disease with no prior treatment
Case 31	tonsil	chronic tonsillitis	benign
Case 32	adenoids	adenoid hyperplasia	benign
Case 33	tonsil	chronic tonsillitis	benign
Case 34	tonsil	chronic tonsillitis	benign
Case 35	tonsil	chronic tonsillitis	benign
Case 36	tonsil	chronic tonsillitis	benign
Case 37	lymph node	melanoma metastasis	Post-surgery Relapse
Case 38	lymph node	Tumor-free lymph node	benign

Some cases were utilized in multiple experiments

Table S4. Summary of Statistics

Plot	Normality test	Distribution	Statistical Tests	p-value	Effect Size Metric	Effect Size	Multiple comparison
Fig. 1B	Shapiro-Wilk test	Non-normal	Kruskal-Wallis test	0.4806	eta-squared (η^2)	0.0276	Dunn's test
Fig. 1D	Shapiro-Wilk test	Normal	RM one-way ANOVA	0.0754	R squared	0.2761	Tukey's test
Fig. 1F	Shapiro-Wilk test	Non-normal	Wilcoxon test	0.3125	Wilcoxon r	1.8	not applicable
Fig. 1H	Shapiro-Wilk test	Normal	Paired t test	0.5904	R squared	0.0786	not applicable
Fig. 1J	Shapiro-Wilk test	Normal	Paired t test	0.6311	R squared	0.0631	not applicable
Fig. 1L	Shapiro-Wilk test	Normal	Paired t test	0.6046	R squared	0.073	not applicable
Fig. 1M	Shapiro-Wilk test	Normal	Unpaired t test	0.0002	R squared	0.777	not applicable
Fig. 1N	Shapiro-Wilk test	Normal	Unpaired t test	0.7894	R squared	0.0075	not applicable
Fig. 1O	Shapiro-Wilk test	Normal	Unpaired t test	0.0125	R squared	0.4804	not applicable
Fig. 1P	Shapiro-Wilk test	Normal	Unpaired t test	0.0108	R squared	0.4938	not applicable
Fig. 1Q	Shapiro-Wilk test	Normal	Unpaired t test	0.1294	R squared	0.2145	not applicable
Fig. 2A	Shapiro-Wilk test	Normal	Paired t test	0.1106	R squared	0.5106	not applicable
Fig. 2B	Shapiro-Wilk test	Normal	Paired t test	0.2997	R squared	0.2616	not applicable
Fig. 2C	Shapiro-Wilk test	Lognormal	Ratio paired t test	0.2132	R squared	0.3536	not applicable
Fig. 2D	Shapiro-Wilk test	Normal	Paired t test	0.3534	R squared	0.2158	not applicable
Fig. 2E	Shapiro-Wilk test	Lognormal	Ratio paired t test	0.0227	R squared	0.7645	not applicable
Fig. 2F	Shapiro-Wilk test	Normal	Paired t test	0.8094	R squared	0.0163	not applicable
Fig. 2G	Shapiro-Wilk test	Normal	Paired t test	0.1246	R squared	0.4844	not applicable
Fig. 2H	Shapiro-Wilk test	Normal	Paired t test	0.2139	R squared	0.3527	not applicable
Fig. 2I	Shapiro-Wilk test	Normal	Paired t test	0.9303	R squared	0.0022	not applicable
Fig. 2J	Shapiro-Wilk test	Normal	Paired t test	0.5402	R squared	0.1006	not applicable
Fig. 2K	Shapiro-Wilk test	Normal	Paired t test	0.0332	R squared	0.7178	not applicable
Fig. 2L	Shapiro-Wilk test	Non-normal	Wilcoxon test	0.625	Wilcoxon r	0.3	not applicable
Fig. 2M	Shapiro-Wilk test	Normal	Paired t test	0.9796	R squared	0.0002	not applicable
Fig. 2N	Shapiro-Wilk test	Normal	Paired t test	0.932	R squared	0.0021	not applicable
Fig. 2O	Shapiro-Wilk test	Normal	Paired t test	0.9672	R squared	0.0005	not applicable
Fig. 2P	Shapiro-Wilk test	Non-normal	Wilcoxon test	0.4375	Wilcoxon r	0.42	not applicable
Fig. 2Q	Shapiro-Wilk test	Normal	Paired t test	0.0553	R squared	0.642	not applicable
Fig. 2R	Shapiro-Wilk test	Normal	Paired t test	0.6425	R squared	0.0591	not applicable
Fig. 4B	Shapiro-Wilk test	Normal	Paired t test	0.0011	R squared	0.9459	not applicable
Fig. 4D	Shapiro-Wilk test	Normal	Paired t test	0.0111	R squared	0.833	not applicable
Fig. 4F	Shapiro-Wilk test	Normal	Paired t test	0.6664	R squared	0.0512	not applicable
Fig. 4I	Shapiro-Wilk test	Normal	Paired t test	0.0023	R squared	0.9694	not applicable
Fig. 4J	Shapiro-Wilk test	Normal	Paired t test	0.9073	R squared	0.0053	not applicable
Fig. 4K	Shapiro-Wilk test	Normal	Paired t test	0.0444	R squared	0.7881	not applicable
Fig. 4L	Shapiro-Wilk test	Normal	Paired t test	0.9097	R squared	0.005	not applicable
Fig. 4M	Shapiro-Wilk test	Normal	Paired t test	0.0416	R squared	0.7965	not applicable
Fig. 5C	Shapiro-Wilk test	Normal	Paired t test	0.013	R squared	0.9742	not applicable
Fig. 5F	Shapiro-Wilk test	Normal	Paired t test	0.0316	R squared	0.8294	not applicable
Fig. 5I	Shapiro-Wilk test	Lognormal	Ratio paired t test	0.0138	R squared	0.6636	not applicable
Fig. 6B	Shapiro-Wilk test	Normal	RM one-way ANOVA	0.0079	R squared	0.9094	Tukey's test
Fig. 6D	Shapiro-Wilk test	Lognormal	Ratio paired t test	0.0121	R squared	0.9086	not applicable
Fig. 6G	Kolmogorov-Smirnov test	Lognormal	Ratio paired t test	0.0009	R squared	0.1353	not applicable
Fig. 6H	Shapiro-Wilk test	Lognormal	Ratio paired t test	0.0783	R squared	0.6975	not applicable
Fig. 6I	Shapiro-Wilk test	Lognormal	Ratio paired t test	0.0092	R squared	0.9234	not applicable
Fig. S2B	Shapiro-Wilk test	Non-normal	Friedman test	0.6543	Kendall's W	0.063	Dunn's test
Fig. S2C	Shapiro-Wilk test	Normal	Paired t test	0.0044	R squared	0.7094	not applicable
Fig. S2D	Shapiro-Wilk test	Normal	Paired t test	0.0001	R squared	0.8913	not applicable
Fig. S2F	Shapiro-Wilk test	Lognormal	Lognormal RM ANOVA	0.0006	R squared	0.8448	Tukey's test
Fig. S2H	Shapiro-Wilk test	Non-normal	Wilcoxon test	0.1562	Wilcoxon r	0.714	not applicable
Fig. S2I	Shapiro-Wilk test	Non-normal	Friedman test	0.5	Kendall's W	0.75	Dunn's test
Fig. S3B	Shapiro-Wilk test	Lognormal	Lognormal RM ANOVA	0.0013	R squared	0.7496	Tukey's test
Fig. S3D	Shapiro-Wilk test	Non-normal	Friedman test	<0.0001	Kendall's W	1	Dunn's test
Fig. S3F	Shapiro-Wilk test	Lognormal	Lognormal RM ANOVA	<0.0001	R squared	0.8933	Tukey's test
Fig. S3H	Shapiro-Wilk test	Non-normal	Friedman test	0.0012	Kendall's W	0.796	Dunn's test
Fig. S3I	Shapiro-Wilk test	Normal	RM one-way ANOVA	0.7467	R squared	0.0928	Tukey's test
Fig. S3J	Shapiro-Wilk test	Non-normal	Friedman test	0.2417	Kendall's W	0.056	Dunn's test
Fig. S3K	Shapiro-Wilk test	Non-normal	Friedman test	0.4321	Kendall's W	0.0375	Dunn's test
Fig. S3L	Shapiro-Wilk test	Normal	RM one-way ANOVA	0.4627	R squared	0.2226	Tukey's test
Fig. S4A	Shapiro-Wilk test	Normal	RM one-way ANOVA	0.0227	R squared	0.9529	not performed
Fig. S4C	Shapiro-Wilk test	Normal	RM one-way ANOVA	0.0209	R squared	0.6854	not performed
Fig. S7A	Shapiro-Wilk test	Normal	RM one-way ANOVA	0.0146	R squared	0.8942	Tukey's test
Fig. S7B	Shapiro-Wilk test	Normal	RM one-way ANOVA	0.004	R squared	0.9255	Tukey's test
